# Magnetic Field Induced Changes in the Shoot and Root Proteome of Barley (*Hordeum vulgare* L.)

**DOI:** 10.3389/fpls.2021.622795

**Published:** 2021-02-23

**Authors:** Azita Shabrangy, Arindam Ghatak, Shuang Zhang, Alfred Priller, Palak Chaturvedi, Wolfram Weckwerth

**Affiliations:** ^1^Molecular Systems Biology Lab, Department of Functional and Evolutionary Ecology, Faculty of Life Sciences, University of Vienna, Vienna, Austria; ^2^VERA Laboratory, Isotope Physics, Faculty of Physics, University of Vienna, Vienna, Austria; ^3^Vienna Metabolomics Center, University of Vienna, Vienna, Austria

**Keywords:** proteomics, barley, magnetic field, geomagnetic field, growth stimulation, root proteome, shoot proteome

## Abstract

The geomagnetic field (GMF) has been present since the beginning of plant evolution. Recently, some researchers have focused their efforts on employing magnetic fields (MFs) higher than GMF to improve the seed germination, growth, and harvest of agriculturally important crop plants, as the use of MFs is an inexpensive and environment-friendly technique. In this study, we have employed different treatments of MF at 7 mT (milliTesla) at different time points of exposure, including 1, 3, and 6 h. The extended exposure was followed by five consecutive days at 6 h per day in barley seeds. The results showed a positive impact of MF on growth characteristics for 5-day-old seedlings, including seed germination rate, root and shoot length, and biomass weight. Furthermore, ~5 days of delay of flowering in pre-treated plants was also observed. We used a shotgun proteomics approach to identify changes in the protein signatures of root and shoot tissues under MF effects. In total, we have identified 2,896 proteins. Thirty-eight proteins in the shoot and 15 proteins in the root showed significant changes under the MF effect. Proteins involved in primary metabolic pathways were increased in contrast to proteins with a metal ion binding function, proteins that contain iron ions in their structure, and proteins involved in electron transfer chain, which were all decreased significantly in the treated tissues. The upregulated proteins' overall biological processes included carbohydrate metabolic process, oxidation-reduction process, and cell redox homeostasis, while down-regulated processes included translation and protein refolding. In general, shoot response was more affected by MF effect than root tissue, leading to the identification of 41 shoot specific proteins. This study provides an initial insight into the proteome regulation response to MF during barley's seedling stage.

## Introduction

The geomagnetic field (GMF) is a natural component of the environment, which acts steadily on living systems during the evolution process, and it is known to influence many biological processes. The GMF's strength at the earth's surface varies from 30 to 70 μT (Maffei, 2014). Many reports suggest that treatment with MFs higher than GMF stimulate the growth and yield of several agronomical essential plant species, although it depends on the flux density and time of exposure of MF (Iqbal et al., 2012; Maffei, 2014). Furthermore, MFs have been shown to modify seed germination and affect seedling growth and development in a wide range of plants, including field, fodder, industrial crops, cereals, and pseudo-cereals (Bhardwaj et al., 2012; Araujo Sde et al., 2016). Static magnetic fields (SMFs) in a range of 30–250 mT (milli Tesla) caused significantly higher values of growth compared to control conditions in important crops like wheat (Payez et al., 2013), maize (Florez et al., 2007), and soya (Baby et al., 2011). In these studies, the attention focused on the physiological effects, including seed germination, growth, development, photosynthesis, and redox status. It was found that MF applied to dormant seeds caused an increase in the rate of subsequent seedling growth in barley, corn (*Zea mays*), beans, wheat, and other tree species (Martinez et al., 2000). Martinez et al. (2000) reported that exposure to 125 mT for different times (1, 10, 20, and 60 min, 24 h, and chronic exposure) stimulated the growth of seedlings especially after 24 h treatment; however, the exposure for a short time (1 min) had a similar effect on growth.

Our previous study demonstrated that EMF treatment with a magnitude of 3 and 10 mT stimulates the growth and development of 7 day-old seedlings of *Zea mays L* and *Brassica napus* and induced oxidative stress (Shabrangi and Majd, 2009; Shabrangi et al., 2010). We also found the induction of genetic variation in EMF pre-treated seedlings compared to control (Shabrangi et al., 2011, 2015). The effects of MFs have been related to the uncoupling of free radical processes in membranes and enhanced ROS generation. MF can change the activities of some scavenging enzymes, such as catalase, superoxide dismutase, glutathione reductase, peroxidase, and polyphenol oxidase, in several plant species (Baby et al., 2011; Rajabbeigi et al., 2013; Aleman et al., 2014; Haghighat et al., 2014).

Barley is a cereal crop mainly used for food, animal feed, and beverage production. Barley is the fourth most important crop in food production after maize, wheat, and rice (FAOSTAT 2016; www.fao.org) (Ghatak et al., 2017; Shabrangy et al., 2018). In our study, we have focused our efforts on using MF to stimulate the growth of cv. Golden promise of barley. Barley seeds rank as the second-largest cereal crop within Europe and account for 19.5% of total cereal production (http://ec.europa.eu/eurostat/; FAOSTAT 2016; www.fao.org). Furthermore, the most frequent use of barley is for malting purposes in the brewing industry and the Golden promise, due to its small grain size, is particularly suited to malt production for distilling (Shabrangy et al., 2018; Orman-Ligeza et al., 2020).

The current study will help to better understand the plant's response at the molecular level to produce better yields using MF-related techniques and leading to the appropriate application of MFs. Here, we have employed a proteomics approach to characterize the barley seedlings under MF with 7 mT flux density for 5 days, 6 h per day. The experimental setup and the data set generated from this study reveal comprehensive physiological and proteomic responses of barley under MF treatment. Thus, this work provides an initial look into the MF responsive proteins in plants and, specifically, in barley.

## Materials and Methods

### Plant Materials

Barley seeds of cv Golden promise were obtained from IPK, Gatersleben, Germany. This cultivar was selected because of the uniform size, shape, and equal average weight of the seeds. Three replicates were used in the experiment with a minimum of 30 seeds in each treatment. The seeds were sterilized with 10% sodium hypo chloride for 5 min and were washed two times with distilled water; they were then placed in the middle of Petri dishes with 100 mm length, and the following two conditions were applied. Condition A: MS medium with 1% agar. Condition B: 1% agar without MS medium. They were placed vertically in the middle of a horizontally-fixed coil. Exposure to MFs was performed using an MF generator. The same conditions were used for control without the power supply.

**Figure 1:**
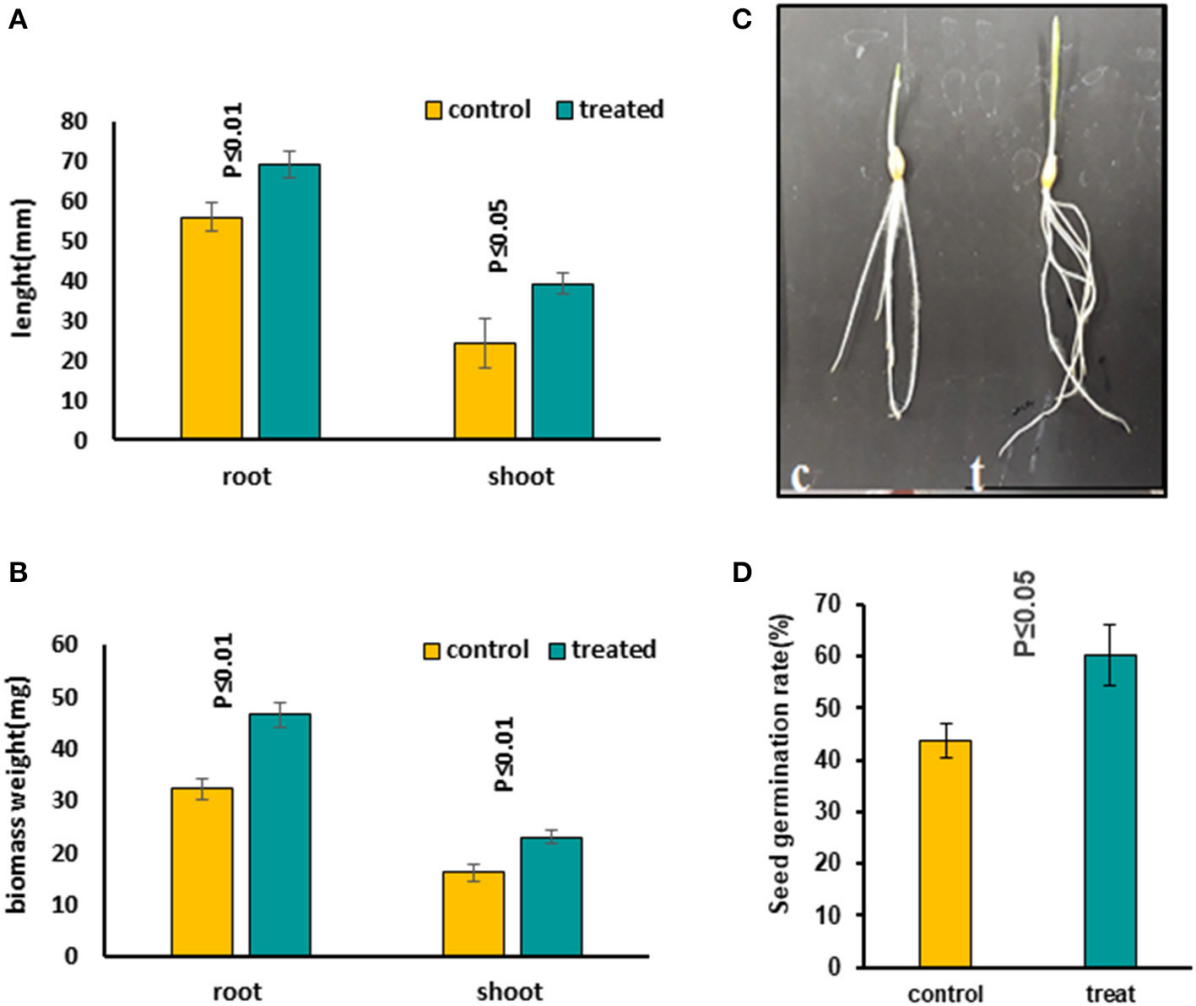
Comparing growth characters in 5-day-old seedlings treated with 7 mT for 6 h per day compared to control seedlings, which were grown on agar without MS medium. **(A)** Root and shoot length increased in treated seedlings compared to the control condition. **(B)** Both root and shoot biomass weight increased significantly in treated seedlings compared to control. **(C)** The figure illustrates a significant growth increase in treated seedlings. **(D)** Seed germination rate increased significantly in treated seeds with MF. Ten to twelve seedlings on average were measured in three independent experiments *P* ≤ 0.05 (c, control; t, treated seedlings with 7 mT for 6 h per day and 5 consecutive day's exposure to MF).

After exposure, Petri dishes were placed in the chamber with a 14–10 h light-dark condition with 60% humidity. The coil's temperature was measured with thermometers to ensure a similar condition with control, which was 22 ± 1°C. All experiments were performed in three biological replicates in three separate Petri dishes. According to our previous study (Shabrangi and Majd, 2009; Shabrangi et al., 2010, 2011), it seems that 7 mT is a suitable flux density to stimulate growth and development for higher plants (Cakmak et al., 2010; Maffei, 2014). Therefore, in this experiment, we have chosen 7 mT of MF generated with a coil and power supply (Figure 1). Seed germination rate and growth characters, including root and shoot length and fresh biomass weight for 5-day-old seedlings, were measured. The differences among the seedlings grown were determined by one-sample *t*-test for three biological replicates, and each replicate contained 30 seeds. We transferred treated seedlings with MF for long exposure and control seedlings to the soil in the glasshouse (the temperature was 18°C and there was a 14/10 h cycle of light-dark; the light was provided at an intensity of 220 μmol photons m^−2^ s^−1^). Harvest index (HI) of the mature plants was calculated according to Ghatak et al. (2016); the plant weight and yield (seed weight) from each replicate were also measured.

#### Magnetic Fields' Exposure Treatment

The seeds were exposed to magnetic fields (MF) produced by a coil manufactured in the lab. A DC power supply (Labornetzgerät QUATPOWER LN-5003XE, Pollin electronic) with adjustable voltage output was used. The cylindrical copper-coil was wound on a polyethene tube, 12 cm in diameter and 50 cm in length (Supplementary Figure 1). Calibration of the system and tests for the accuracy and uniformity of the MF was performed using a Gauss-meter with a Hall Effect based B-probe (HIRS Magnetic Instruments Ltd., Type GM08). The points used for the following equation are measured with both Transverse Probe (type TP002) and Axial Probe (AP002). A diagram of the data is shown in Supplementary Figure 1B. The equation for calculating the MF strength is:

$$\text{B}\left(\text{I}\right)=0.014\text{ mT}+5.245\;\frac{\text{mT}}{\text{A}}\text{I}$$

where *B* is the MF strength measured in mT, and I is the current passed through the coil measured in Ampere. The electric field generated by the voltage applied to the coil can be estimated as follows. The coil has four layers on the plastic tube. Only the inner layer is seen by the seeds since there is no gap between the windings. For maximum voltage applied (50 V) and a length 0.5 m of the coil, there was an electric field of 50 V/4/0.5 m = 25 V/m parallel to the magnetic field. To get an MF of 7 mT, about 40 V had to be applied to the coil. The resulting electrical field strength was then 20 V/m. The temperature recorded with a thermometer read 22 ± 1°C. We applied 7 mT of MF for different time points including 1, 3, and 6 h and a long exposure for 5 days, 6 h a-day. With no MF applied, untreated seeds were used as control under similar conditions. Statistical analysis was done using the student *t*-test.

### Proteomics Extraction and LC-MS/MS Analysis

All samples (5-day-old seedlings) were frozen in liquid nitrogen to stop any enzymatic activity and were further subjected to proteomic analysis. MF leads to redistribution of the cellular activities. This is why applying the proteomic analysis to the whole plant is not very informative (Herranz et al., 2013; Maffei, 2014). Therefore, in this study, we separated the root and shoot from the 5-day-old seedlings for further analysis.

Protein extraction, pre-fractionation, digestion, and LC-MS/MS analysis was performed according to previous studies (Chaturvedi et al., 2013, 2015; Valledor and Weckwerth, 2014). Root and shoot tissues were harvested and ground with mortar and pestle. The homogenized samples were suspended in 200 μl of protein extraction buffer (100 mM Tris–HCl, pH 8.0; 5% SDS, 10% glycerol; 10 mM DTT; 1% plant protease inhibitor cocktail (Sigma P9599) and incubated at room temperature for 5 min, followed by incubation for 2.5 min at 95°C and centrifugation at 21,000 × g for 5 min at room temperature. The supernatant was transferred carefully to a new tube. Two hundred microliters of 1.4 M sucrose were added to the supernatant and proteins were extracted twice with 200 μl TE buffer equilibrated phenol followed by counter extraction with 200 μl of 1.4 M sucrose and 200 μl distilled water. Phenol phases were combined and subsequently mixed with 2.5 volumes of 0.1 M ammonium acetate in methanol for precipitation of proteins. After 16 h of incubation at −20°C, samples were centrifuged for 5 min at 5,000 × g. The pellet was washed twice with 0.1 M ammonium acetate, once with acetone, and then air-dried at room temperature. The pellet was re-dissolved in 6 M urea and 5% SDS, and protein concentration were determined using the bicinchoninic acid assay (BCA method). Pre-fractionation of proteins was carried out by SDS-PAGE (Paul et al., 2016; Jegadeesan et al., 2018). Forty micrograms of total protein were loaded onto a gel and run for 1.5 cm. Gels were fixed and stained within Methanol: Acetic Acid: Water: Coomassie Brilliant Blue R-250 (40:10:50:0.001), destained in methanol: water (40:60), and then each lane was divided into two fractions. Gel pieces were destained, equilibrated, digested with trypsin Sequencing Grade (Roche 11418 475 001), desalted, and then concentrated. Prior to mass spectrometric measurement, the tryptic peptide pellets were dissolved in 4% (v/v) acetonitrile and 0.1% (v/v) formic acid. One μg of each sample (three biological replicates samples) was loaded on a C18 reverse-phase column (Thermo scientific, EASY-Spray 500 mm, 2 μm particle size). Separation was achieved with a 90 min gradient from 98% solution A (0.1% formic acid) and 2% solution B (90% ACN and 0.1% formic acid) at 0 min to 40% solution B (90% ACN and 0.1% formic acid) at 90 min with a flow rate of 300 nL min^−1^. nESI-MS/MS measurements were performed on Orbitrap Elite (Thermo Fisher Scientific, Bremen, Germany) with the following settings: Full scan range 350–1,800 m/z resolution 120,000 max. 20 MS2 scans (activation type CID), repeat count 1, repeat duration 30 s, exclusion list size 500, exclusion duration 30 s, charge state screening enabled with the rejection of unassigned and +1 charge states, minimum signal threshold 500.

### Data Processing and Protein Identification

Raw files were processed with MaxQuant 1.6 (http://www.maxquant.org) and the Andromeda search algorithm (Tyanova et al., 2015). Barely UniProt database was used for the identification of proteins. Peptide identification was performed using the following settings: mass tolerance for precursors was set to 5 ppm and for fragment masses up to 0.8 Da. The maximum FDR was set to 0.01%. Three missed cleavages were allowed. The variable modifications were set to oxidation of methionine (M) and protein N-terminal acetylation. There were no fixed modifications, as dynamic modifications were used. Label-free quantification (LFQ) was done at the MS1 level with at least two peptides per protein. PTXQC was used to assess data quality (Roustan et al., 2018). All the identified proteins were searched for the closest Arabidopsis (Tair 10) and wheat orthologs using stand-alone BLAST v2.2.28+ (using the default matrix) in conjunction with an unpublished Python script used for the following homology searches. The top three hits with an e-value below the threshold of 10^−3^ were selected from the results for further comparison. All the differential proteins were also functionally annotated according to biological process, cellular component, and molecular function using Uniprot (http://www.uniprot.org) and DAVID (http://david.abcc.ncifcrf.gov/) (Supplementary Tables 2–4). The Venn diagram was produced by using Venny (http://bioinfogp.cnb.csic.es/tools/venny/index.html). All raw data and result files (annotated spectra) are uploaded to ProteomeXchange. Submission details are as follows: Project name: Magnetic field induced changes in the proteomic analysis in barley (*Hordeum vulgare*). Project accession: PXD022079.

### Statistical Evaluation of Data

To avoid miscalculation of missing values, statistics were only performed on the set of proteins present in all three biological replicates. We used LFQ (label-free quantification) intensities, which were calculated by MaxQuant. Protein annotation was performed using the MERCATOR tool (http://mapman.gabipd.org/de). The sum of the normalized LFQ intensities for each functional category is represented by bi-cluster using the statistical toolbox Perseus and COVAIN in Matlab (Sun and Weckwerth, 2012). Functional annotation of the unknown proteins was identified using BLAST at the UniProt homepage, searching for the closest homolog in the cereal protein dataset. The obtained data were first Log_2_ transformed, and the normalized dataset was used to calculate the relative protein abundance. Multivariate analysis (PCA), one-way analysis of variance (ANOVA), and Student's *t*-tests were performed using Perseus 1.6.5.0 software and significantly changed proteins between control and MF treatment were further considered (Ott et al., 2019). The generation of protein-protein interaction (PPI) networks was conducted via the Search Tool for the Retrieval of Interacting Genes/Proteins (STRING) database for known and predicted protein-protein interactions (http://string-db.org/) with default parameters (Franceschini et al., 2013).

## Results

### Seedling Growth Characteristics in Response to MF With Different Exposure Times

We evaluated the influence of MFs with different exposure times on seeds to find out the most effective treatment. Several MF flux densities and exposure times were examined (Shabrangi et al., 2010, 2011). The optimized treatment was found to be 7 mT, which caused a significant increase in seed germination rate, accelerating the root emergence, with growth stimulation in the seedling including root and shoot length and fresh biomass of barley (Figures 1A–D). Then growth characters of 5-day-old seedlings exposed to an MF of 7 mT at different time points, including 1, 3, and 6 h, and a long exposure for 5 days, 6 h per day, were measured. An increase in length and weight of both root and shoot tissues in all these treatments was observed (Supplementary Figures 2A–G) and the significant increases occurred when seeds were continuously exposed to the 7 mT for 5 days, 6 h per day (Figures 1A–D). Therefore, for further studies, we took long exposed samples. The following conditions were applied when preparing these samples. In condition A, seeds were exposed to MF on MS medium. Prolonged exposure to MF caused a decrease of seed germination rate and growth characteristics, including root and shoot length and their fresh biomass weight in all plants (Supplementary Figures 3A–E). Short exposure of seeds in MS medium with the same flux density of MF (7 mT) for 6 h exposure caused growth inhibition of seedlings, which was less prominent than in long exposure (Supplementary Figures 4A–D). In condition B, seeds were exposed to MF on agar without MS medium. Prolonged exposure to the same flux density of MF (7 mT) caused increased seed germination rate, accelerated root emergence, and stimulated growth characters of seedlings, including root and shoot length and their fresh biomass weight in all plants (Figures 1A–D). A significant decrease in growth was observed in the MS medium's seedlings (Supplementary Figures 3, 4). Contrastingly, an increased growth in the long-term treatment with the same condition on the agar without MS medium was observed (Figures 1A–D).

**Figure 2:**
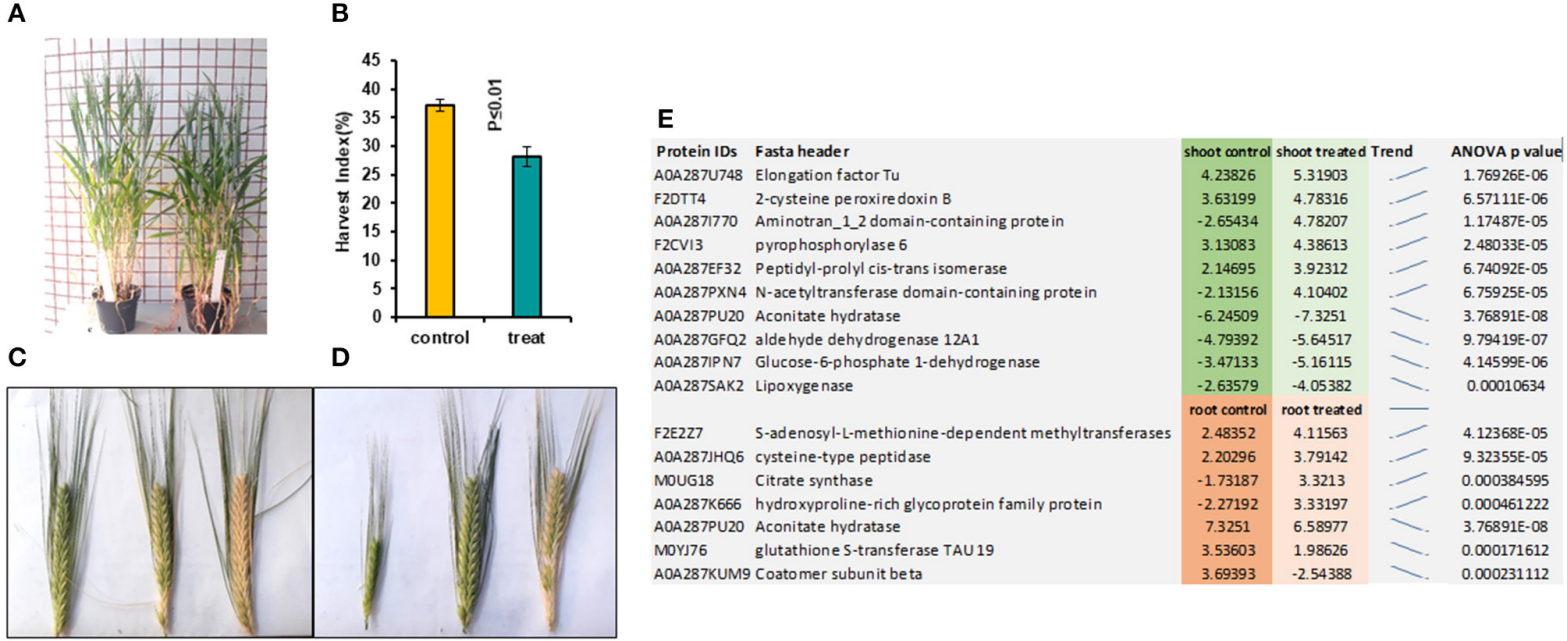
This figure illustrates pre-treated plants with 7 mT for 6 h per day for 5 consecutive days and control plants. **(A)** Pre-treated plants showed 5 days' delay in flowering compared to control. **(B)** Harvest index in pre-treated plants decreased significantly compared to control. **(C)** Ears harvested from the same control plant show similar times of developmental stages. **(D)** Ears harvested from the same pre-treated plants showed a variety of different developmental stages. **(E)** The highest significantly changed proteins in both roots and shoot tissues compared to control.

In the later stage of plants grown from pre-treated seedlings with 7 mT for 6 h per day and 5 consecutive days' exposure, we observed a delay in flowering in the treatment compared to control (Figure 2A). We also observed a significant decrease of Harvest Index (HI) in pre-treated plants with MF compared to control plants (Figure 2B). Furthermore, varying developmental stages of seeds were observed in the pre-treated plants with MF (Figure 2D). In contrast, seeds of control plants from different ears per plant showed almost similar seed development stages (Figure 2C).

### Tissue-Specific Comparative Proteome Analysis

**Figure 3:**
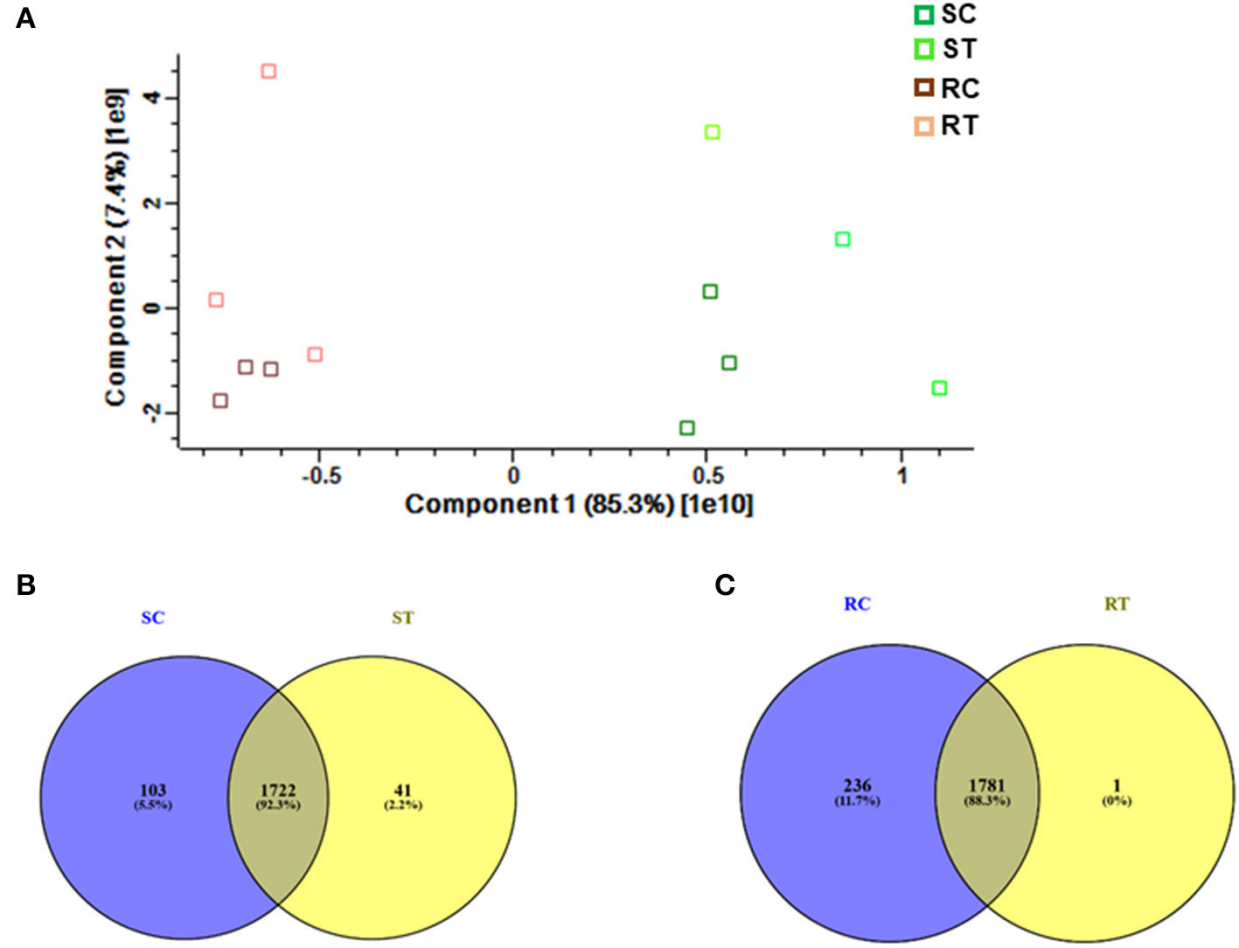
**(A)** Proteome profiling in 5-day-old seedlings of barley, which were exposed to 7 mT for 6 h per day for 5 consecutive days. Principal Component Analysis (PCA) was conducted on logarithmically transformed protein LFQ; each dot corresponds to a single biological replicate (*n* = 3). Venn diagram illustrates, **(B)** Number of common and specific proteins between control and treated shoot tissue, **(C)** Number of common and specific proteins between control and treated root tissue (RT, root treated; RC, root control; ST, shoot treated; SC, shoot control).

Comparative proteomic analysis was performed using an LC-MS/MS technique (Ghatak et al., 2016, 2021). Both shoot and root tissues of 5-day-old seedlings of barley after prolonged exposure to 7 mT (condition B) and control samples were considered to identify putative proteome signatures associated with MF response. By using label-free quantitative proteomics profiling, we identified a total of 4,906 proteins. After normalization of the data, 2,896 proteins were quantified, of which 349 were significantly changed among different treatments and tissues (Supplementary Table 1, Supplementary Figure 5). Results were based on one-way analysis of variance (ANOVA) and *P*-value ≤ 0.01 was considered significantly different, tests were performed with Perseus 1.5 software. All the identified proteins were subject to BLAST to identify the closest homolog by using the UniProt homepage searching among the cereal protein database. We have identified significantly changed proteins, which potentially explain the response mechanism of the plant against MF. The principal component analysis (PCA) underlines pronounced changes in the tissue-specific proteome, grouping samples in a tissue-specific and treatment-specific manner (Figure 3A).

### Shoot Proteome Under MF Treatment

We identified 1,722 proteins in common between both conditions (control and treatment). In shoot tissue, 38 proteins were identified, which significantly changed between control and treated condition (Table 1, Figure 3B). Detailed information about these proteins is provided in Supplementary Table 2.

**Table 1:** Details of significantly different proteins under MF effects in shoots of 5-day-old seedlings (NA, Unknown).

**Protein ID**	**Description**	**Log_**2**_ Ratio MF/Controls**	**ANOVA (*p* value)**	**No. of peptides**	**Identification confidence**	**Function**	**Arabidopsis orthologs**	**Wheat orthologs**
A0A287GFQ2	Aldehyde dehydrogenase 12A1	1.18	0.01	15	69.87	Encodes mitochondrial Delta-pyrroline-5- carboxylate dehydrogenase and involved in the catabolism of proline to glutamate	NA	AAL70109
A0A287U748	Elongation factor Tu	1.26	0.01	22	323.31	EF-Tu is responsible for catalyzing the binding of an aminoacyl-tRNA to the ribosome	AT4G20360	AQU14666
A0A287IPN7	Glucose-6-phosphate 1-dehydrogenase	1.49	0.01	18	137.66	glucose metabolic process	AT3G27300	BAA97663
F2DTT4	2-cysteine peroxiredoxin B	1.32	0.01	11	79.56	Encodes a 2-Cys peroxiredoxin (2-Cys PrxB) that contains two catalytic Cys residues	AT5G06290	SPT18356
A0A287I770	Aminotran-1-2 domain containing protein	−1.81	0.01	14	71.9	Identified by cloning the gene that corresponded to a purified protein having glyoxylate aminotransferase activity	AT1G23310	SPT17977
F2CVI3	Pyrophosphorylase 6	1.41	0.01	11	94.97	Encodes a protein with inorganic pyrophosphatase activity.	AT5G09650	AFK26595
A0A287EF32	Peptidyl-prolyl cis-trans isomerase	1.83	0.01	6	60.93	PPIases accelerate the folding of proteins and catalyzes the cis-trans isomerization of proline imidic peptide bonds in oligopeptides	AT5G13120	ABO37960
A0A287PXN4	N-acetyltransferase domain-containing protein	−1.93	0.01	3	40.02	The locus shares considerable sequence similarity with the adjacent NATA1 gene. They encode genes with different functions.	AT2G39020	NA
A0A287SAK2	Lipoxygenase	1.54	0.0001	50	323.31	Plant lipoxygenase may be involved in a number of diverse aspects of plant physiology	AT1G55020	ACS34908
F2DUQ7	Pyridoxine biosynthesis protein	1.74	0.0001	7	69.89	Encodes a protein functions in tandem with PDX2 to form glutamine amidotransferase complex that involved in vitamin B6 biosynthesis.	AT2G38230	AAZ94411
F2E7T5	Succinate dehydrogenase [ubiquinone] iron-sulfur subunit, mitochondrial	1.77	0.0001	7	46.1	Iron-sulfur protein (IP) subunit of succinate dehydrogenase (SDH) involves in complex II of the mitochondrial electron transport chain.	AT5G40650	CAD62367
A0A287L8J4	Pyruvate kinase	−0.56	0.0002	9	123.51	Catalytic activity, ATP + pyruvate = ADP + H+ + phosphoenolpyruvate	AT1G32440	CDM82744
M0XGG9	Actin 7	−1.12	0.0006	19	14.68	Member of Actin gene family and mutants are defective in germination and root growth	AT5G09810	AHE76167
A0A287T3T5	CHAPERONIN-60BETA2, CPN60BETA2, CPNB2	−0.68	0.0007	20	86.65	Encodes a subunit of chloroplasts chaperonins involved in mediating the folding of newly synthesized, translocated, or stress-denatured proteins.	AT3G13470	AHH93014
A0A287V7D1	Aconitate hydratase	−1.69	0.0008	19	121.76	Encodes an aconitase that can catalyze the conversion of citrate to isocitrate through a cis-aconitate intermediate	AT2G05710	ADD62592
A0A287S1D0	Triosephosphate isomerase	−1.83	0.0009	11	81.66	Encodes triosephosphate isomerase.	AT3G55440	CAC14917
A0A287L247	PNP-UDP-1 domain-containing protein	−1.96	0.001	6	118.56	PNP_UDP_1 domain-containing protein	AT4G28940	NA
F2CWX3	Allene oxide synthase	−0.53	0.002	22	90.98	Encodes a member of the cytochrome p450 CYP74 gene family that functions as an allene oxide synthase.	AT5G42650	AHM92934
M0X5Z1	Translation initiation factor eIF-4A1	−1.18	0.002	21	171.92	Eukaryotic translation initiation factor 4A-1	AT3G13920	AQU14667
A0A287NU21	RNAse inhibitor protein2	−1.69	0.003	7	143.09	member of RLI subfamily	AT4G19210	AAL26702
A0A287N7A0	Endoglucanase	−0.59	0.003	3	13.4	Catalytic activity, Endohydrolysis of (1->4)-beta-D-glucosidic linkages in cellulose, lichenin and cereal beta-D-glucans.	AT4G16260	AAM13693
M0XII1	Peptidyl-prolyl cis-trans isomerase	−1	0.003	12	72.7	PPIases accelerate the folding of proteins and catalyzes the cis-trans isomerization of proline imidic peptide bonds in oligopeptides.	AT2G29960	ACF49500
A0A287H7K0	Hydroxyproline-rich glycoprotein family protein	−1.96	0.004	9	82.12	Encodes a nucleocytoplasmic lectin that is capable of binding carbohydrates.	AT1G04930	AFN10736
M0YIA7	Isocitrate dehydrogenase (NADP)	−1	0.005	17	252.08	Encodes a NADP+-isocitrate dehydrogenase that contributes to NADPH production under oxidative stress.	AT1G65930	AMP82030
A0A287H7K2	Hydroxyproline-rich glycoprotein family protein	−1.33	0.005	6	62.73	Encodes a nucleocytoplasmic lectin that is capable of binding carbohydrates.	AT1G04930	AFN10736
A0A287PSF7	Heat shock protein 60	−1.57	0.006	27	102.51	Mitochondrial chaperonin HSP assist in rapid assembly of the oligomeric protein structures in the mitochondria.	AT3G23990	AHH93014
A0A287VBD4	DUF3357 domain-containing protein	−1.68	0.006	9	43.64	Encodes a vacuolar invertase betaFruct4 that is transported from the endoplasmic reticulum through the intermediate compartments as a membrane protein.	AT1G12240	AMZ79593
A0A287WLE3	3-hydroxy-3-methylglutaryl coenzyme A synthase	−0.78	0.008	8	34.58	This enzyme condenses acetyl-CoA with acetoacetyl-CoA to form HMG-CoA, which is the substrate for HMG-CoA reductase.	AT4G11820	NA
A0A287WPQ1	60S ribosomal protein L27	−1.04	0.01	6	72.16	Ribosomal L27e protein family	AT4G15000	3J61_Z
F2DIR3	40S ribosomal protein S4	−0.76	0.02	14	81.56	Ribosomal protein S4 (RPS4A) family protein	AT5G58420	3J60_E
A0A287UJD3	Inorganic pyrophosphotase	−1.25	0.02	3	19.47	Encodes a soluble protein with inorganic pyrophosphatase activity that is highly specific for Mg-inorganic pyrophosphate.	AT3G53620	AFK26595
A0A287Q0B2	Uncharacterized protein	−1	0.02	9	55.04	Encodes an enzyme with ATPase and ADPase activity (an apyrase)	AT5G18280	SPT15600
A0A287MY92	dUTPase domain-containing protein	−1	0.03	6	30.02	DUTP-PYROPHOSPHATASE-LIKE 1	AT3G46940	CDM82885
A0A287GTQ9	V-type proton ATPase subunit C	−1	0.03	20	140.01	Subunit of the peripheral V1 complex of vacuolar ATPase that is responsible for acidifying a variety of intracellular compartments in eukaryotic cells	AT1G12840	ABG23314
A0A287TGY1	Calnexin 1	−1	0.03	15	148.65	Calnexin 1	AT5G61790	AGF69181
A0A287KMB8	Reticulon-like protein	−1	0.04	4	22.22	Reticulan like protein B3; (source:Araport11)	AT1G64090	CDJ26559
A0A287PP23	Late embryogenesis abundant domain-containing protein /LEAdomain-containing protein	−0.92	0.007	15	83.83	Late embryogenesis abundant domain-containing protein/LEA domain-containing protein	NA	AAA34267

**Figure 4:**
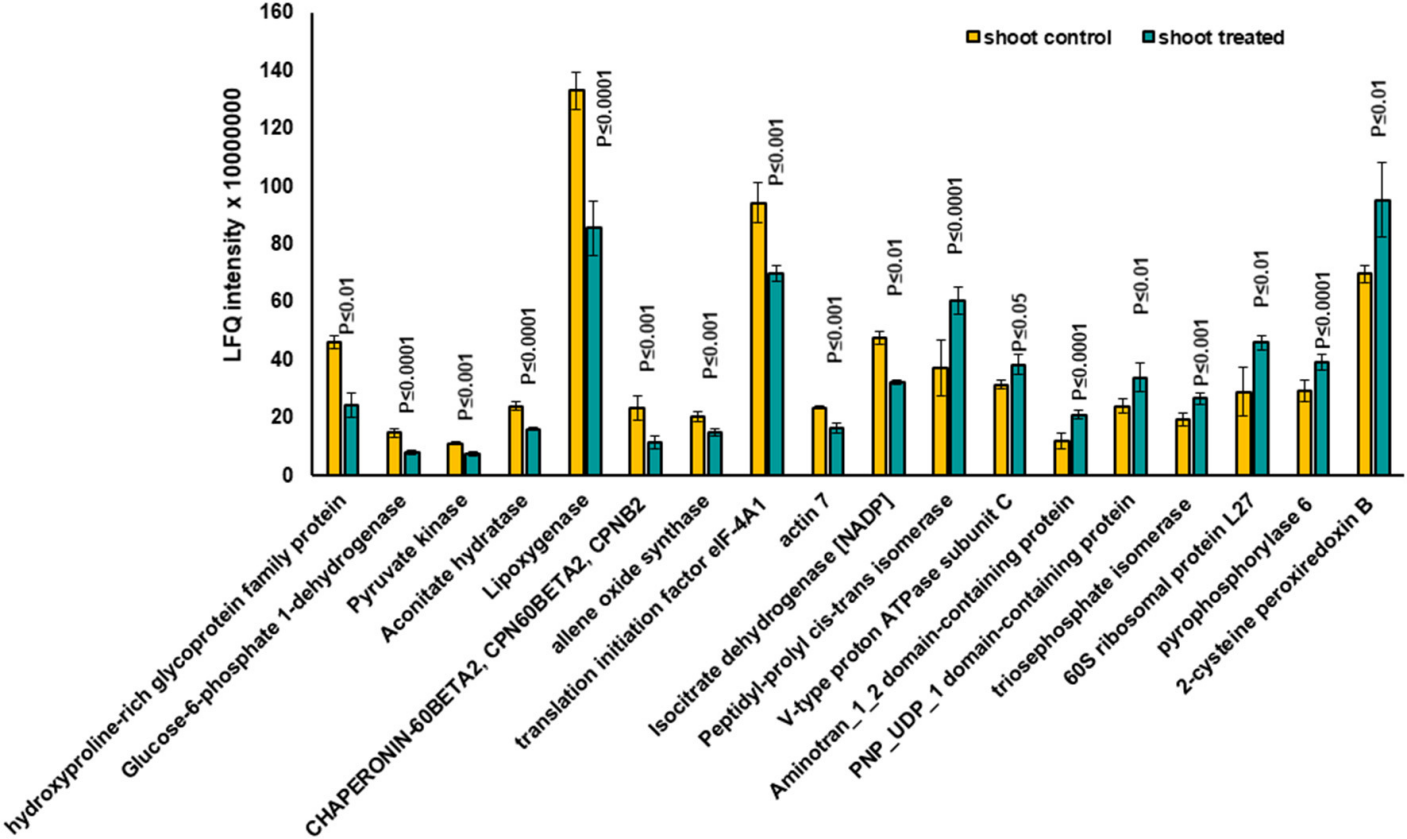
The diagram illustrates the most significantly changed proteins in shoot tissue treated with 7 mT for 6 h per day over 5 consecutive days.

Based on the PCA, proteins with the highest positive loadings showed highest abundance under control conditions, including RNAse inhibitor protein 2, calnexin, and allene-oxide synthase, with metal ion binding function, which showed a significant decrease in treated shoot tissue compared to control (Table 1, Figure 4). We identified the abundance level of proteins such as pyruvate kinase and isocitrate dehydrogenase with magnesium ion binding function, which decreased significantly in treated shoot tissue compared to control. These proteins are located in the chloroplast and are involved in photosynthesis. Magnesium is an integral component of chlorophyll and serves an essential function for photosynthesis (Sharma et al., 2018).

Proteins with high abundance under the effects of MF (Table 1, Figure 4) include phosphorylase superfamily protein, pyrophosphorylase 1, and pyrophosphorylase 6 with inorganic diphosphatase activity.

V-type proton ATPase subunit C was another abundant protein identified in the shoots under MF effect with proton transmembrane transporter activity. Similarly, a group of proteins with catalytic and folding activities was identified, including alanine-2-oxoglutarate aminotransferase 2, peptidyl-prolyl cis-trans isomerase, and 60S ribosomal protein L27 which showed an increased level of abundance in treated shoot tissue.

Significantly changed proteins in MF treated shoot tissue were localized in the endomembrane compartments, including endoplasmic reticulum (ER) (42%), Golgi apparatus (34%), and vacuoles (28.9%). Proteins such as reticulon-like protein, calnexin1, and peptidyl-prolyl cis-trans isomerase were localized in both ER and Golgi apparatuses (Supplementary Table 2). Some of these proteins, such as calnexin 1, were located in different organelles of the endomembrane system (Supplementary Table 2). Therefore, these data suggested that intracellular trafficking in the shoot might be rearranged under the influence of MF (Supplementary Table 2). The highest significantly changed proteins in shoot tissues compared with control are illustrated in Figure 2E.

**Figure 5:**
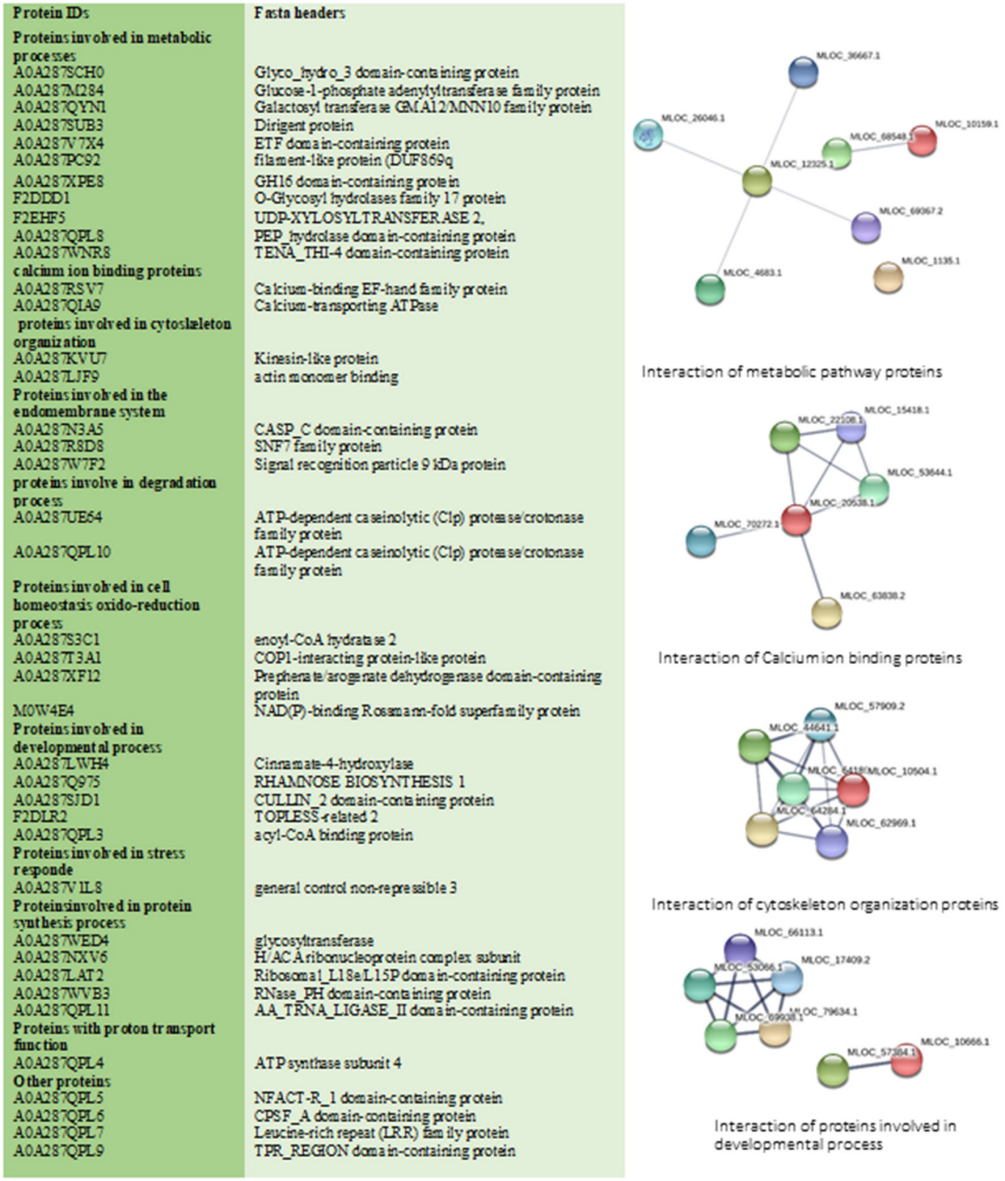
This figure illustrates all specific treated shoot proteins categorized in different groups. Changed proteins were analyzed by STRING database. STRING default parameters were used (https://string-db.org/). Interactions of proteins in each group are shown.

In total 41 proteins were identified to be putative MF responsive proteins unique to treated shoot tissue (Figure 3B, Supplementary Table 3). Among these, four proteins were identified with metal ion function, including cytochrome b, calcium-transporting ATPase, glutamine aminotransferase type-2 domain-containing protein, and cinnamate-4-hydroxylase (Figure 5). The molecular function of these identified proteins is iron-binding (Petit et al., 2001). Cinnamate-4-hydroxylase is involved in several biological processes, which include developmental processes (Yadav et al., 2019), lignin metabolism (Shigeto et al., 2013), oxidation-reduction, phenylpropanoid biosynthetic, phenylpropanoid metabolic, and ubiquinone biosynthetic processes (Bell-Lelong et al., 1997). Another protein, known as actin monomer binding with actin-binding function and actin 7 protein, was also identified with increased levels in treated shoots compared to shoots in the control condition, which suggests cytoskeleton involvement in response to MF (Supplementary Table 3, Figure 5). Recent studies indicate the crucial influence of external mechanical and magnetic forces on the cell shape, function, and fate through physical interactions with the cytoskeleton network (Zablotskii et al., 2016).

The identified 41 proteins varied in their subcellular localization: 36.6% were located in the endomembrane compartment, 22% in mitochondria, and 24.5% in the chloroplast (detailed information in Supplementary Tables 2, 3). Calcium-binding EF-hand family protein and 2-cysteine peroxiredoxin were identified as shoot-specific proteins under MF treatment (Figure 5). Protein 2-cysteine peroxiredoxin B contains two catalytic Cys residues and is located in the apoplast, chloroplast, cytoplasm, and plasma membrane. It has peroxiredoxin activity and it is involved in several biological cell processes such as cell redox homeostasis, defense response to the bacterium, and response to oxidative stress (Figure 5) (Liebthal et al., 2018). In general, MF applied in our study initiated a stress response mechanism, which is evident from the peroxiredoxin's increased levels.

### Root Proteome Under MF Treatment

**Figure 6:**
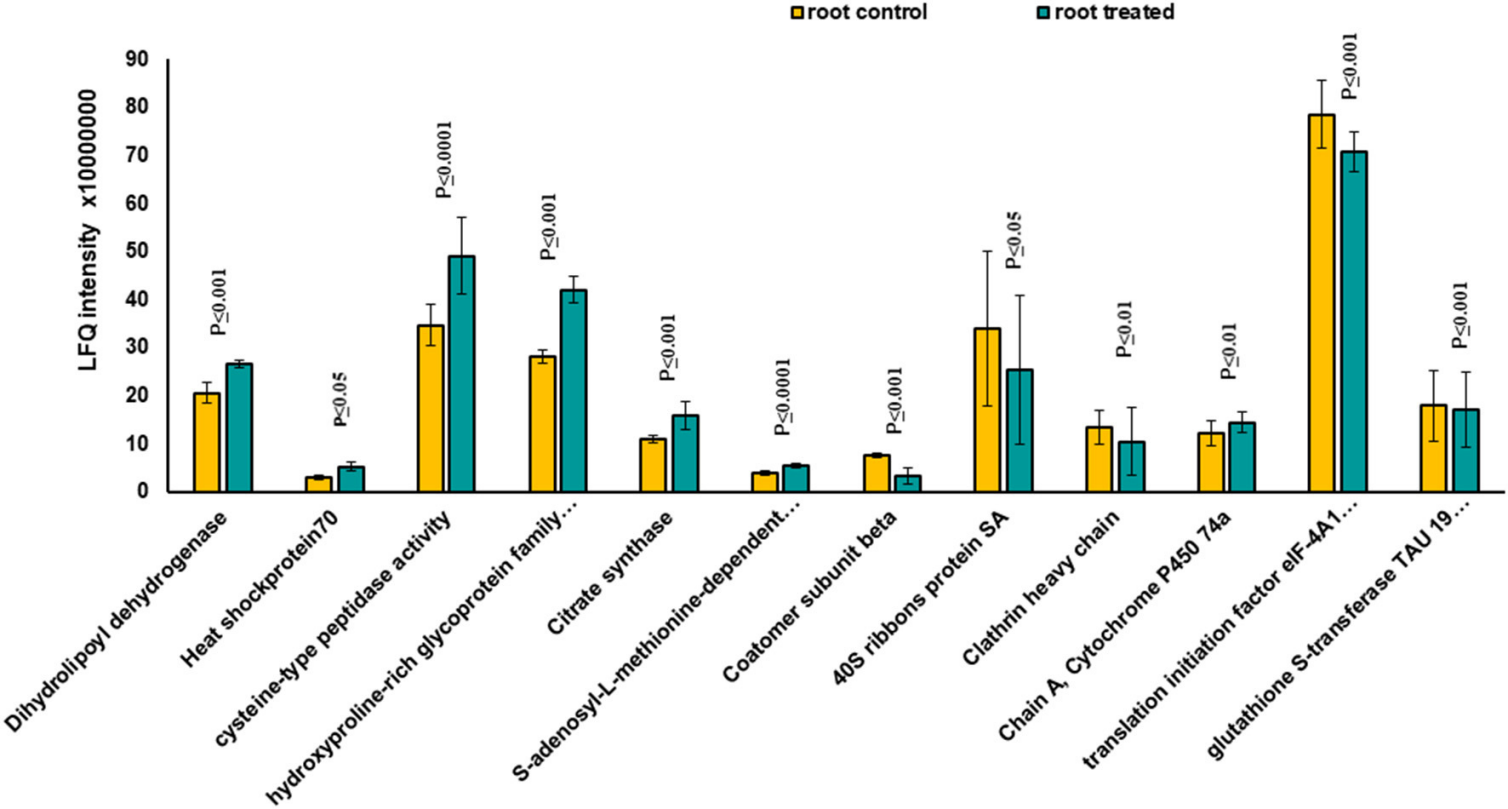
The diagram illustrates all significantly changed proteins in root tissue treated with 7 mT for 6 h per day over 5 consecutive days.

Based on Venn analysis (Figure 3C), from all the detected peptides, 1,781 proteins were identified in root under both the control and MF treatment, of which 236 proteins were identified only in the control condition (Figure 3C). Interestingly, no unique proteins were identified in the MF-treated root tissue. Several proteins were identified as significantly different between the conditions (controls and MF treatment) (Figure 6, Table 2; Supplementary Table 4). Proteins with metal ion binding functions, such as clathrin heavy chain, aconitate hydratase, cytochrome P450, and 40S ribbons protein SA, decreased significantly in treated root compared to control (Figure 6). Another protein, coatomer subunit beta, which is an intracellular protein transport and a vesicle-mediated transport, with a synonym of copper-induced intracellular protein transport (Ortiz-Zapater et al., 2006), also decreased significantly under treated root (Figure 6). Proteins with higher abundance in treated roots compared to control include citrate synthase, which is involved in crucial biological cell processes, including tricarboxylic acid cycle (TCA) and dihydrolipoyl dehydrogenase with cell redox homeostasis. All significantly changed proteins between control and treated roots are illustrated in Supplementary Table 4 with LFQ flux density.

**Table 2:** Details of significantly different proteins in roots of 5-day-old seedlings treated with MF (NA, not known).

**Protein IDs**	**Description**	**Log_**2**_ Ratio MF/Controls**	**ANOVA (*p* value)**	**No.of peptides**	**Identification confidence**	**Function**	**Arabidopsis orthologs**	**Wheat orthologs**
A0A287KUM9	Coatomer subunit beta	−0.69	0.0002	19	99.89	Intracellular protein transport, vesicle-mediated transport	AT1G79990	CDM82914
A0A287MUK5	Uncharacterized protein	−0.69	0.002	10	56.47	NA	NA	NA
A0A287PQY8	40S ribosomal protein SA	−1	0.03	6	38	40S ribbons protein SA	AT3G04770	AQU14669
A0A287PU20	Aconitate hydratase	0.9	0.01	27	236.78	Catalyzes the isomerization of citrate to isocitrate via cis-aconitate	AT4G26970	ADD62592
A0A287R3A1	Clathrin heavy chain	−0.5	0.002	39	323.31	Clathrin coat assembly, intracellular protein transport, vesicle-mediated transport	AT3G08530	CDM83157
F2CWX3	Cytochrome P450 74a	−2.22	0.002	22	90.98	Encodes a member of the cytochrome p450 CYP74 gene family that functions as an allene oxide synthase	AT5G42650	AHM92934
M0X5Z1	Translation initiation factor eIF-4A1	−0.6	0.002	21	171.92	Translation initiation factor eIF-4A1	AT1G53880	AQU14667
M0YJ76	Glutathione S-transferase TAU 19	0.56	0.0002	11	54.3	Encodes a glutathione transferase that is a member of Tau GST gene family. Expression is induced by drought stress, oxidative stress, and high doses of auxin and cytokinin	AT1G78380	CAC94002, ACE82289
A0A287EXJ0	Dihydrolipoyl dehydrogenase	−2.14	0.001	15	121.25	Cell redox homeostasis, Oxidoreductase	AT3G17240	AGX45478.1
A0A287G6G3	Heat shockprotein70	−1	0.02	22	42.41	Heat shockprotein70	AT3G12580	AAB99745
A0A287JHQ6	Cysteine-type peptidase	1.72	0.01	5	88.48	Cysteine-type peptidase activity	AT2G07240	AWW05355
A0A287K666	Hydroxyproline-rich glycoprotein family protein	−1.47	0.000461	14	110.97	Late embryogenesis abundant hydroxyproline-rich glycoprotein family protein	AT2G39050	AFN10736
M0UG18	Citrate synthase	−1.92	0.0003	11	137.59	Tricarboxylic acid cycle	AT4G34050	AMP82031
F2E2Z7	S-adenosyl-L-methionine-dependent methyltransferases superfamily protein	1.66	0.01	11	33.95	S-adenosyl-L-methionine-dependent methyltransferases superfamily protein	AT1G01180	BAD06321

Interestingly, both treated root and shoot proteomes showed a decrease in the abundance of proteins with a metal ion binding function, and in proteins containing metal ion in their structure. In contrast, proteins involved in primary metabolic pathways increased in both treated tissues compared to control (Tables 1, 2). It indicates that shoot tissue response was more vital to MF treatment than root tissue. Hence, it can be concluded that MF has a different impact on different tissues.

### Hierarchical Cluster Analysis Illustrating Significantly Changed Proteins in Different Tissues Under MF

Hierarchical cluster analysis of significant proteins under control and treated conditions was performed with Perseus after the data's z-score transformation (Supplementary Figure 5; Supplementary Table 1). Clustering of proteins was done based on Euclidian distance while sample clustering was based on Pearson correlation. Three hundred forty-nine proteins were found significantly changed between different tissues (root and shoot) and different conditions (Controls and MF treatment). All these differential proteins are detailed in Supplementary Table 1. Cluster analysis revealed a specific group of proteins, either changing concentrations in tissue or MF treatment.

**Figure 7:**
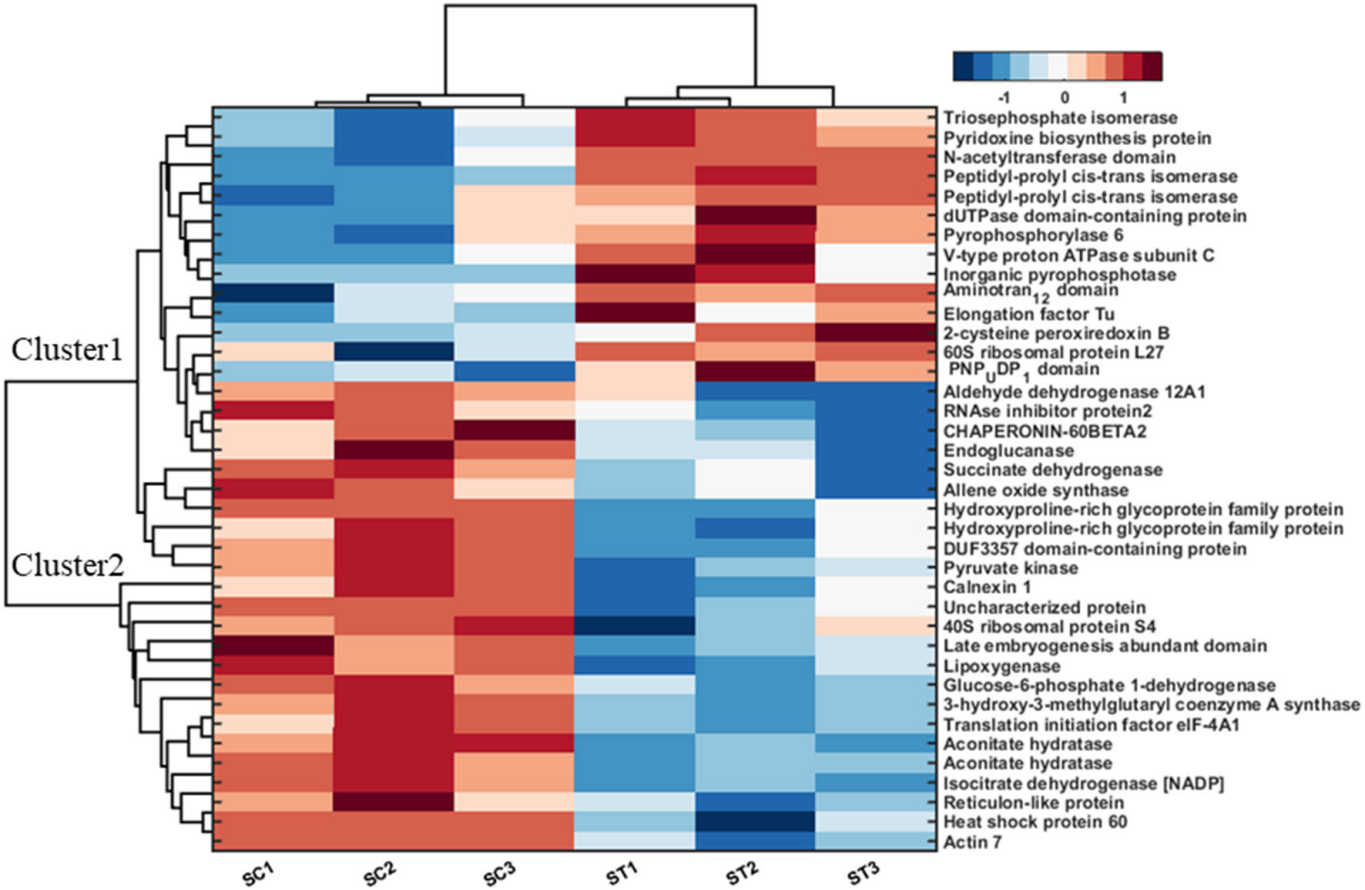
Bi-cluster analysis of molecular functional protein categories in shoots, which significantly changed under control and MF exposure conditions (7 mT for 6 h per day for 5 consecutive days). All identified proteins were categorized into functional groups to allow for a functional view of the treated-specific proteome. The sum of the normalized spectral abundance factor for each functional category was analyzed by bi-clustering using the statistical toolbox COVAIN. The bi-clustering uses the average linkage of Euclidean distance between groups as the metric (SC, Shoot control; ST, Shoot treated).

Further, 38 differentially expressed proteins in shoot tissue were represented using bi-cluster analysis in COVAIN in Matlab (Sun and Weckwerth, 2012). Here, two clusters were identified (Figure 7). Fourteen proteins were grouped in cluster I and 24 proteins in cluster II. The abundance of the proteins in cluster I increased after exposure to MF, while proteins in cluster II decreased in abundance after exposure to MF (Figure 7). These proteins were likely to be active during a specific process. The upregulated proteins were involved in overall biological processes, including metabolic processes, especially carbohydrate metabolic process, proton transport, oxidation-reduction process, and cell redox homeostasis.

In contrast, down-regulated proteins were mostly involved in translational initiation, translation, and protein refolding (Supplementary Table 2). The most upregulated proteins were located in the cytosol, cytoplasm, and chloroplast endomembrane compartment, while down-regulated proteins were assigned to the ribosome (Supplementary Table 2). The molecular functions of upregulated proteins were related to motor and enzymatic activity, including diphosphatase and isomerase activity. In contrast, down-regulated proteins were involved in metal ion binding functional proteins, binding activity, and dehydrogenase activity proteins (Supplementary Table 2).

## Discussion

MFs are considered as non-ionizing radiation that can positively influence several plants' morphogenesis, which allows them to be used as an environmentally friendly and non-toxic stimulus for seed germination and growth of plants. Some studies have shown that MFs and EMFs between 1.5 and 250 mT had a positive effect on seed germination and seedling growth in different plant species, and enhanced the biomass and yield (Vashisth and Nagarajan, 2010; Cakmak et al., 2012; Naz et al., 2012). In this study, an overall stimulating effect on growth characteristics of 5-day-old seedlings observed under 7 mT for different time points of the exposure, including 1, 3, and 6 h as a treatment (Supplementary Figure 2) and for 5 consecutive days at 6 h per day. The highest growth observed was for the most prolonged exposure of 5 consecutive days, 6 h per day in barley seeds grown on agar without MS medium (Figures 1A,D). Ghaffari et al. (2016) reported that weight accumulation is correlated with ADP-glucose pyrophosphorylase activity (AGPase), a rate-limiting enzyme involved in starch biosynthesis in barley. Similarly, we observed a significant increase in the biomass weight of treated seedlings with MF (Figure 1B and Supplementary Figure 2). This can also be correlated to the proteomics data that showed increased levels of pyrophosphorylase. Interestingly, pre-sowing magnetic treatment of soybean was found to improve biomass accumulation (Shine et al., 2011). In the previous study, *Zea mays* and *Brassica napus* under electromagnetic fields (EMFs) treatment showed an overall stimulating effect on the growth of seedlings and increase of biomass weight (Shabrangi et al., 2010, 2011).

A significant difference in the growth of the seedlings was observed with MS medium (Supplementary Figures 3, 4) and without MS medium (Figures 1A,D). This difference could be due to the change in ion absorption under MF effects. Narayana et al. reported a general reduction of *Arabidopsis* roots' ion uptake and transport. It eventually also affected the plant's growth and development immediately after exposure to the reduced MF (Narayana et al., 2018). The accumulation of metals in plant tissues, mainly iron and zinc content, were affected under near null magnetic field (NNMF) condition and iron uptake genes were induced in the roots of NNMF-exposed plants (Islam et al., 2020). Under iron deficiency, NNMF-exposed plants displayed a limitation in the activation of iron-deficiency-induced genes, leading to the substantial accumulation of zinc and copper. Further, it was concluded that GMF could contribute to efficient iron uptake in *Arabidopsis thaliana* (Islam et al., 2020).

Based on BLAST results, more than 50% of significantly changed proteins with their tissue-specific abundance could be putative candidates for MF, localized in the plasma membrane and specifically in the endomembrane compartment (Supplementary Table 2). Zhao reported that the cell membrane is an important site of interaction for shallow frequency (ELF) EMFs. The permeability of ions can be changed by EMFs, which is a theoretical model of cytosolic calcium oscillations influenced by MFs (Zhao et al., 2008). Moreover, MF causes mechanical stress in the membrane (Zablotskii et al., 2016), as we observed many significant proteins are membrane-localized in response to MF.

Some proteins with calcium ion binding function increased in treated shoot tissues, such as calcium-binding EF-hand family proteins. MFs could change the ion permeability of cell membranes. A theoretical model of cytosolic calcium oscillations influenced by MFs has been reported previously (Galland and Pazur, 2005; Zhao et al., 2008). The increase in cytosolic Ca^2+^ after exposure to weak MF suggested that Ca^2+^ entry into the cytosol and Ca^2+^ homeostasis can constitute the primary weak MF sensing mechanism in plants (Belyavskaya, 2004; Galland and Pazur, 2005).

**Figure 8:**
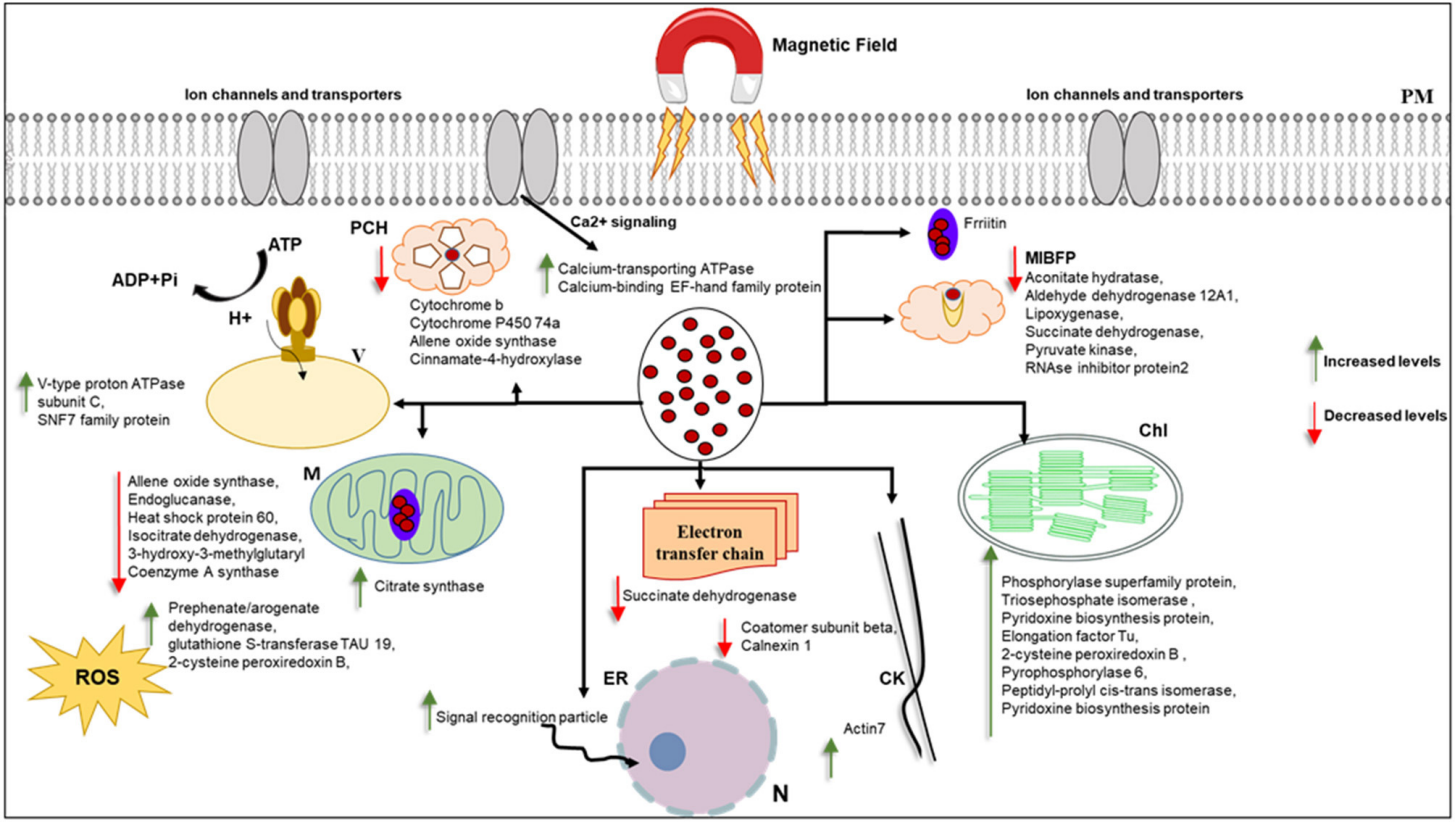
This figure hypothetically represents the MF effects on different organelles via significantly changed proteins. MF effects on electron transport chain and consequently photosynthesis and metabolism of the cell. Iron as a ferromagnetic element directly affects the ferritin and molecules containing heme in their structure. Schema is not in scale, N, nucleus; ER, endoplasmic reticulum; M, mitochondria; MIBF, metal ion binding function proteins; PCH, proteins contain Heme; PM,plasma membrane; Chl, Chloroplast; ROS, reactive oxygen species; V, vacuole; CW, cell wall; CK, cytoskeleton.

Most of the proteins that responded to MF showed significant changes in shoot tissue compared to roots (Table 1, Supplementary Table 2). Transition metals are essential micronutrients for all living forms, principally due to their ability to couple with proteins to form metalloproteins (Bowman et al., 2016). Iron metal has an incomplete “d” orbital, which permits it to attain different oxidation states and redox chemistry. As a result of this unique chemistry, iron acts as a cofactor for several enzymes and has essential roles in oxygen metabolism, electron transport, tricarboxylic acid (TCA) cycle, lipid metabolism, and peroxide detoxification (Krewulak and Vogel, 2008; Cornelis et al., 2011; Ma et al., 2015). Iron, as a ferromagnetic metal ion, has a large, positive susceptibility to external MF (Figure 8). Iron exhibits a strong attraction to MFs and can retain their magnetic properties after the external field has been removed due to unpaired electrons, so their atoms have a net magnetic moment (Sharma et al., 2018). Ferritin is a universal intracellular protein that stores iron and releases it in a controlled fashion. Vaezzadeh et al. (2006) proposed a theoretical model based on ferritin's oscillation, the iron-storage protein in ferritin cells when exposed to external MF. It seems that, due to the oscillation of metal ions in the cell, after exposure to MF applied in this study, proteins with metal ion binding function decreased in treated tissues of barley (Figure 8).

Cytochrome b is an essential protein involved in mitochondrial electron transport with metal ion binding function and involvement in the oxidation-reduction process, found in the MF-treated shoot (Turek et al., 2014). Since most biological substances are proteins that contain metal ions, such as hemoglobin, cytochrome, or ferritin, they are paramagnetic. The paramagnetic (Fe and Co content) and diamagnetic (starch) components differed when exposed to different static MFs and forces (Vaezzadeh et al., 2006). Belyavskaya (2004) also reported the reduction of phyto-ferritin in plastids of meristem cells in pea roots when exposed to weak MF.

The shoot specific protein cinnamate 4-hydroxylase (CYP73 or CA4H) is a member of the P450 superfamily. The molecular function of this protein is iron-binding, and it is involved in different cell biological processes, such as the lignin metabolic process (Schilmiller et al., 2009). According to the previous study, lignification in xylem vessels of both roots and shoot tissue was observed in pre-treated seedlings with EMFs in *Zea mays* and *Brassica napus*, which could be due to the higher activity of peroxidase enzyme that is involved in lignin synthesis (Shabrangi and Majd, 2009).

It is worth noting that several studies have documented ROS involvement in seed germination (Oracz et al., 2007; Jeevan Kumar et al., 2015). Oracz et al. reported that sunflower seeds treated with methyl-viologen, an ROS-generating compound, led to the release of dormant seeds and caused the oxidation of a specific set of embryo proteins. They proposed a mechanism for seed dormancy alleviation, which involves ROS production and targeted changes in protein carbonylation patterns (Oracz et al., 2007). ROS accumulation is essential in breaking seed dormancy and stimulating seed germination and protection from pathogens (Jeevan Kumar et al., 2015). MFs have been shown to increase the oscillation and concentration of free oxygen radicals in cells, increasing cell stress and thus cells' response through the production of antioxidant enzymes (Shabrangi and Majd, 2009; Rajabbeigi et al., 2013; Haghighat et al., 2014). We observed the stimulation and increase of seed germination rate of barley under MF used in this study. Similarly, in the proteome analysis, we also observed an increased abundance of proteins with oxide-reduction activity, such as aldehyde dehydrogenase, in response to MF (Figure 8).

Actin7, involved in cell division, cytoskeleton organization, response to auxin, response to light stimulus, response to wounding, root development, and seed germination, showed a decreased abundance in treated shoots compared to control (Kijima et al., 2018). According to the proteomics data, the cytoskeleton may respond to MF, as demonstrated in this study (Figure 8).

We also observed biomass increases in treated seedlings with 7 mT MF, which could be due to the increased abundance of proteins involved in primary metabolic pathways. MF may cause stimulating effects on metabolism during seed germination and seedling growth. Chloroplasts contain Mg^2+^, which is a paramagnetic substance and plays an essential role in photosynthesis. When an external MF is applied to Mg^2+^ it tends to move into the MF direction. This interaction absorbs energy that could affect chloroplasts, disturb pigment synthesis, and affect photosynthesis and biomass production (Zhang et al., 2018).

Further, UDP-XYLOSYLTRANSFERASE2 identified among specific shoot proteins treated with MF can add several xylosyl residues to the acceptor forming mono-, di- and trixylosylated polysaccharides (Cavalier and Keegstra, 2006), which can be involved in biomass increase under MF condition. The proteomics data showed many significantly changed proteins under MF that are located in chloroplasts (Supplementary Tables 2–4). Racuciu et al. (2008) reported growth stimulation of maize seedlings under 50 mT treatment, and chlorophyll content increased significantly, but it decreased as MF flux density increased from 50 up to 250 mT. MF effects on chlorophyll content have been reported for several other plant species (Turker et al., 2007; Radhakrishnan and Ranjitha Kumari, 2012). Applied MF in our study also caused growth stimulation in barley seedlings and the delay of flowering in the later stage (Figure 2C). Xu et al. reported a delay in flowering in pre-treated Arabidopsis with NNMF. The biomass accumulation of plants in the NNMF was significantly suppressed when plants were switching from vegetative growth to reproductive growth compared with that of plants grown in the local GMF (Xu et al., 2013; Agliassa et al., 2018).

A significant decrease in the HI of the pre-treated plants compared to control was observed. Similar results were also reported by Xu et al., (Figure 2D). They observed a significant reduction of 20% in the harvest index of plants in the NNMF compared to the controls, and we observed 22.7% of HI reduction in pre-treated plants with MF compared to control (Figure 2D). Furthermore, proteome analysis demonstrated an increased level of ETF domain-containing protein as a specific shoot-treated protein. Mutations of the ETF beta gene result in accelerated senescence (Ishizaki et al., 2005); these results are consistent with the delay of flowering in pre-treated plants.

Interestingly, proteome data revealed the stimulating effects of MF in this study on primary metabolic pathways, consequently growth and developmental alteration in seedlings, and a delay of flowering in the later stage of growth. At the same time, HI showed a significant decrease in pre-treated plants with MF. However, it can be concluded that MF induced metabolism alteration in the young seedlings. Simultaneously, the effect is suppressed in the later stages of plant development by showing lower HI in the pre-treated plants.

Exposure of seedlings to NNMF causes a decrease in the seedling growth (Belyavskaya, 2004; Binhi and Prato, 2017). Higher flux density applied in this study caused stimulation in the growth and development of seedlings. Hence it can be concluded that MF can be used as a natural biotechnological tool to stimulate seed germination, growth, and development of the plant.

## Conclusion

Our data revealed that exposure to 7 mT for 6 h and 5 consecutive days caused stimulating effects on the growth and development of seedlings, which could be due to stimulating primary metabolic pathways. According to the proteomics data, shoot tissue responds stronger to MF than root tissue in the early developmental stage of plant growth. The main question that remains is “How will the cell respond and adapt to a MF?” MF effects could be due to the interplay between biological and physical factors in the cell machinery. Proteomics data revealed that MF causes changes in the regulation of proteins with a metal ion binding function, whereas other proteins involved in primary metabolic pathways increased in abundance. The data suggested that MF would cause changes in cytoskeleton remodeling, and it could have a potential impact on the proteins involved in cell homeostasis, oxidoreduction activity, and catalytic activity. The proteomics data, especially in shoot tissue, suggested that intracellular trafficking is rearranged during MF exposure. This study provides a first reference data set for future investigations and a comprehensive view of the proteins potentially involved in response to MF during the early stage of growth and development in barley. Furthermore, some of the significantly changed proteins under MF should be attractive priorities for our future studies.

## Data Availability Statement

The datasets presented in this study can be found in online repositories. The names of the repository/repositories and accession number(s) can be found below: http://www.proteomexchange.org/, PXD022079.

## Author Contributions

AS and AG conceived and designed the experiments. AS and AP designed and set up the MF generation. AS wrote the manuscript. AS and SZ performed the protein extraction. AG and PC performed LC-MS/MS analysis. AS, AG, PC, and WW analyzed the proteomics data and statistics. PC, AG, and WW critically read and edited the manuscript. All authors read and approved the final version of the manuscript for publication.

## Conflict of Interest

The authors declare that the research was conducted in the absence of any commercial or financial relationships that could be construed as a potential conflict of interest.

## Supplementary Material

Supplementary Figure 1 MF set up, **(A)** figure of coil connected to a power supply with DC current, **(B)** diagrams illustrate the ratio of current produced by power supply in the coil and the flux density of MF generated in the coil.

Supplementary Figure 2 This figure illustrates 5-day-old barley seedlings exposed to 7 mT for different time points grown on agar without medium. All growth parameters increased in treated seedlings. **(A)** 0 h exposure to MF. **(B)** 1 h exposure to MF. **(C)** 3 h exposure to MF. **(D)** 6 h exposure to MF. **(E)** Seed germination rate (%) after treatment with 7 mT for different exposure times on agar without medium. **(F)** Root and shoot length of seedlings, treated with 7 mT for different time points compared with control samples. **(G)** Fresh biomass weight of 5-day-old treated seedlings with 7 mT for different exposure times (Mean value of three biological replicates, *P* ≤ 0.05 based on student *t*-test).

Supplementary Figure 3 The figure illustrates 5-day-old seedlings' growth characters in response to 7 mT for 6 h per day for 5 consecutive days that were grown on MS medium. **(A)** Control seedlings. **(B)** Treated seedlings. **(C)** Seed germination rate (%) increased in treated seeds. **(D)** Root and shoot length decreased significantly in response to long exposure to MF. **(E)** Fresh biomass weight of seedlings treated with MF for long exposure showed a significant decrease (c, control; t, treated).

Supplementary Figure 4 The figure illustrates 5-day-old seedlings' growth characters in response to 7 mT for 6 h that were grown on MS medium. **(A)** Control seedlings. **(B)** Treated seedlings. **(C)** Root and shoot length decreased significantly in response to long exposure to MF. **(D)** Fresh biomass weight of seedlings treated with MF for long exposure showed a significant decrease.

Supplementary Figure 5 Hierarchical cluster analysis of all significantly changed proteins in different tissues including roots and shoots of 5-day-old seedlings and different conditions, including control and those treated with 7 mT for 5 days, 6 h a day (RT, root treated; RC, root control; ST, shoot treated; SC, shoot control).

Supplementary Table 1 Detailed information of all significantly changed proteins in abundance between different tissues (shoot and root) and different conditions (treatment with MF and control condition) in 5-day-old seedlings treated with 7 mT for 5 days, 6 h a day.

Supplementary Table 2 Detailed information of significantly changed proteins in abundance under MF effects in shoot tissue compared to shoot control condition of 5-day-old seedlings. All the differential proteins were also functionally annotated according to biological process, cellular component, and molecular function (NN, not known).

Supplementary Table 3 Detailed information of specific shoot proteins under MF effects in 5-day-old seedlings. All specific shoot proteins were also functionally annotated according to biological process, cellular component, and molecular function (NN, not known).

Supplementary Table 4 Detailed information of significantly changed proteins in abundance under MF effects in root tissue compared to the root control condition of 5-day-old seedlings. All the differential proteins were also functionally annotated according to biological process, cellular component, and molecular function (NN, not known).
