# Supplementary material for: Magnetic Field Induced Changes in the Shoot and Root Proteome of Barley (Hordeum vulgare L.)
Source: Front Plant Sci. 2021 Feb 23;12:622795. doi: 10.3389/fpls.2021.622795 (PMC7940674; doi:10.3389/fpls.2021.622795)
Supplement: Supplementary Figure 1 — MF set up, (A) figure of coil connected to a power supply with DC current, (B) diagrams illustrate the ratio of current produced by power supply in the coil and the flux density of MF generated in the coil. [file Data_Sheet_1.ZIP › Supplementary figures.pptx]

## Slide 1
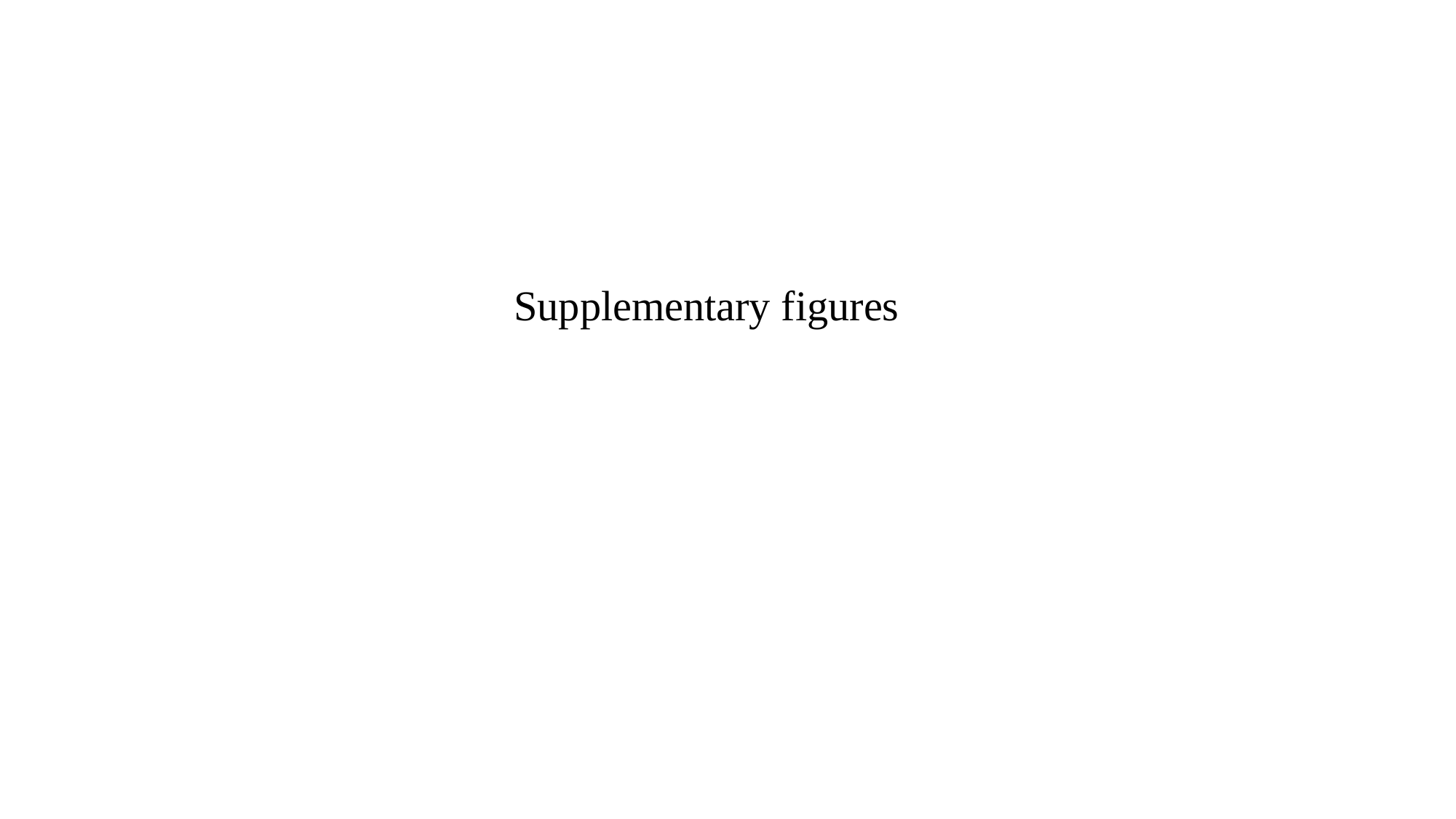

Supplementary figures

## Slide 2
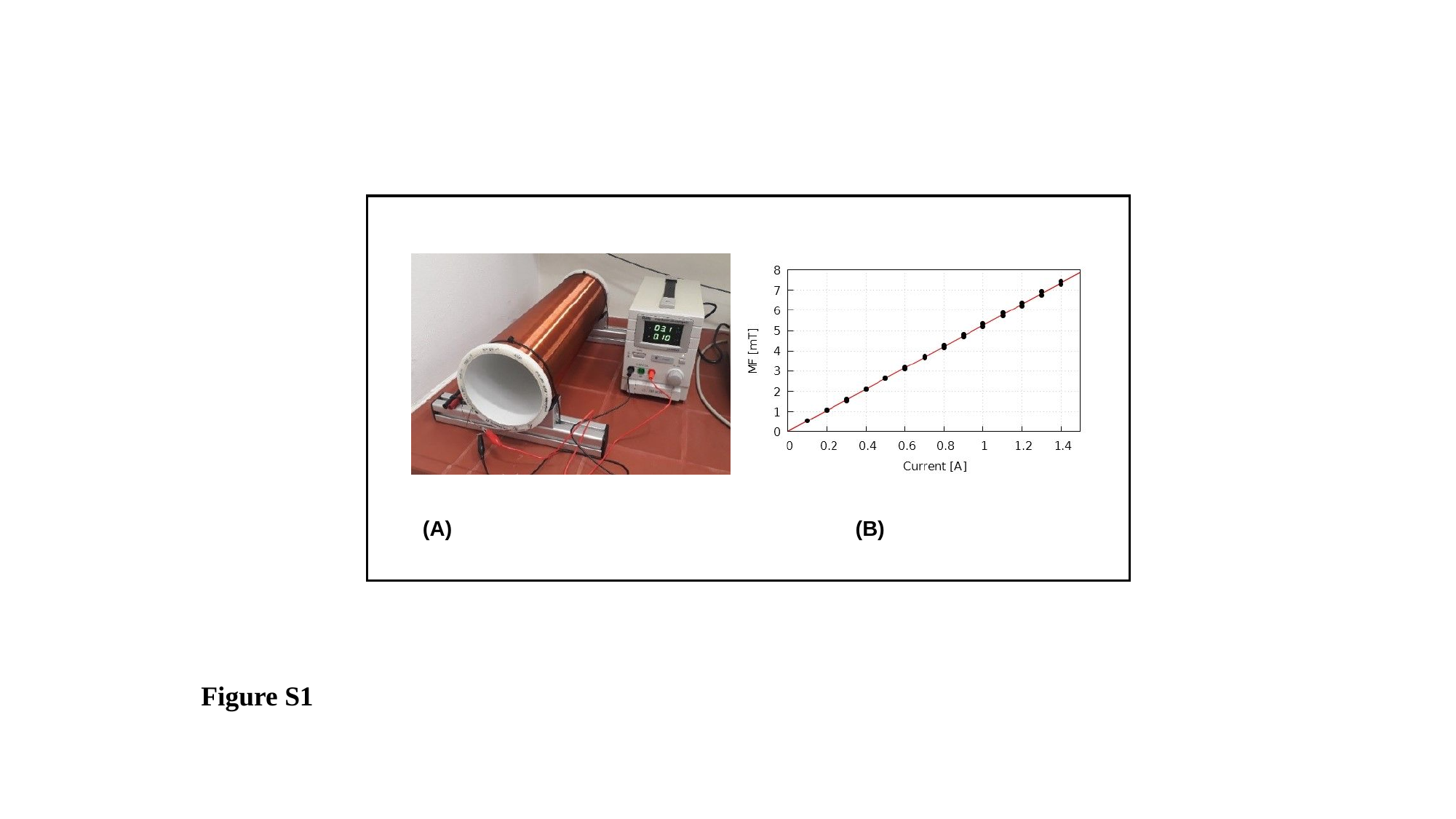

(A)
(B)
Figure S1

## Slide 3
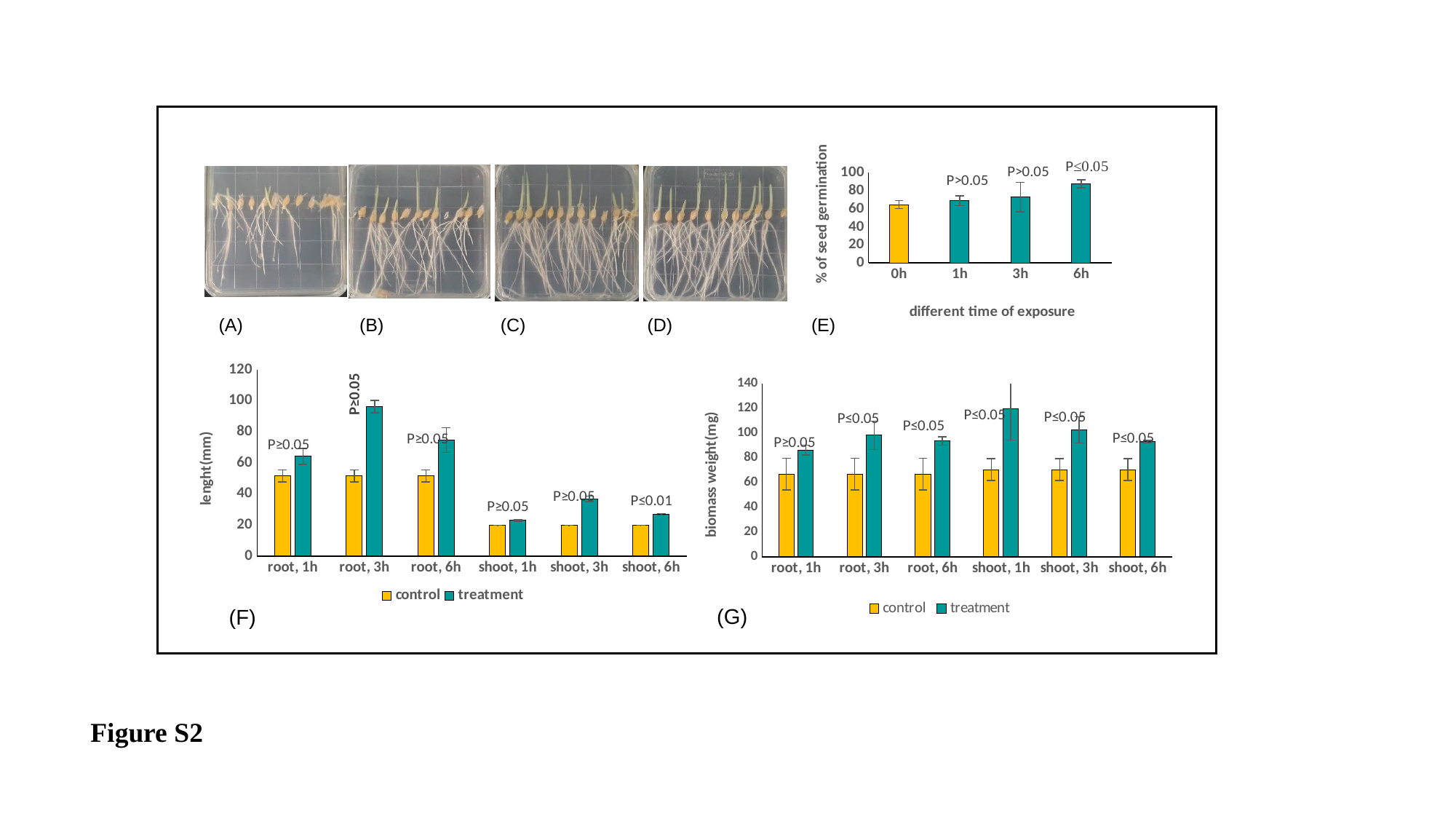

### Chart:
| Category | seed germination rate |
|---|---|
| 0h | 64.6 |
| 1h | 68.85 |
| 3h | 73.05 |
| 6h | 87.75 |
 (A) (B) (C) (D) (E)
### Chart
| Category | control | treatment |
|---|---|---|
| root, 1h | 51.53 | 64.1 |
| root, 3h | 51.53 | 96.2 |
| root, 6h | 51.53 | 74.6 |
| shoot, 1h | 19.8 | 22.94 |
| shoot, 3h | 19.8 | 36.8 |
| shoot, 6h | 19.8 | 26.84 |
### Chart
| Category | control | treatment |
|---|---|---|
| root, 1h | 66.84444444444445 | 86.26666666666667 |
| root, 3h | 66.84444444444445 | 98.18055555555554 |
| root, 6h | 66.84444444444445 | 93.57 |
| shoot, 1h | 70.33611111111111 | 119.67222222222222 |
| shoot, 3h | 70.33611111111111 | 102.5222222222222 |
| shoot, 6h | 70.33611111111111 | 93.008 |(G)
(F)
Figure S2

## Slide 4
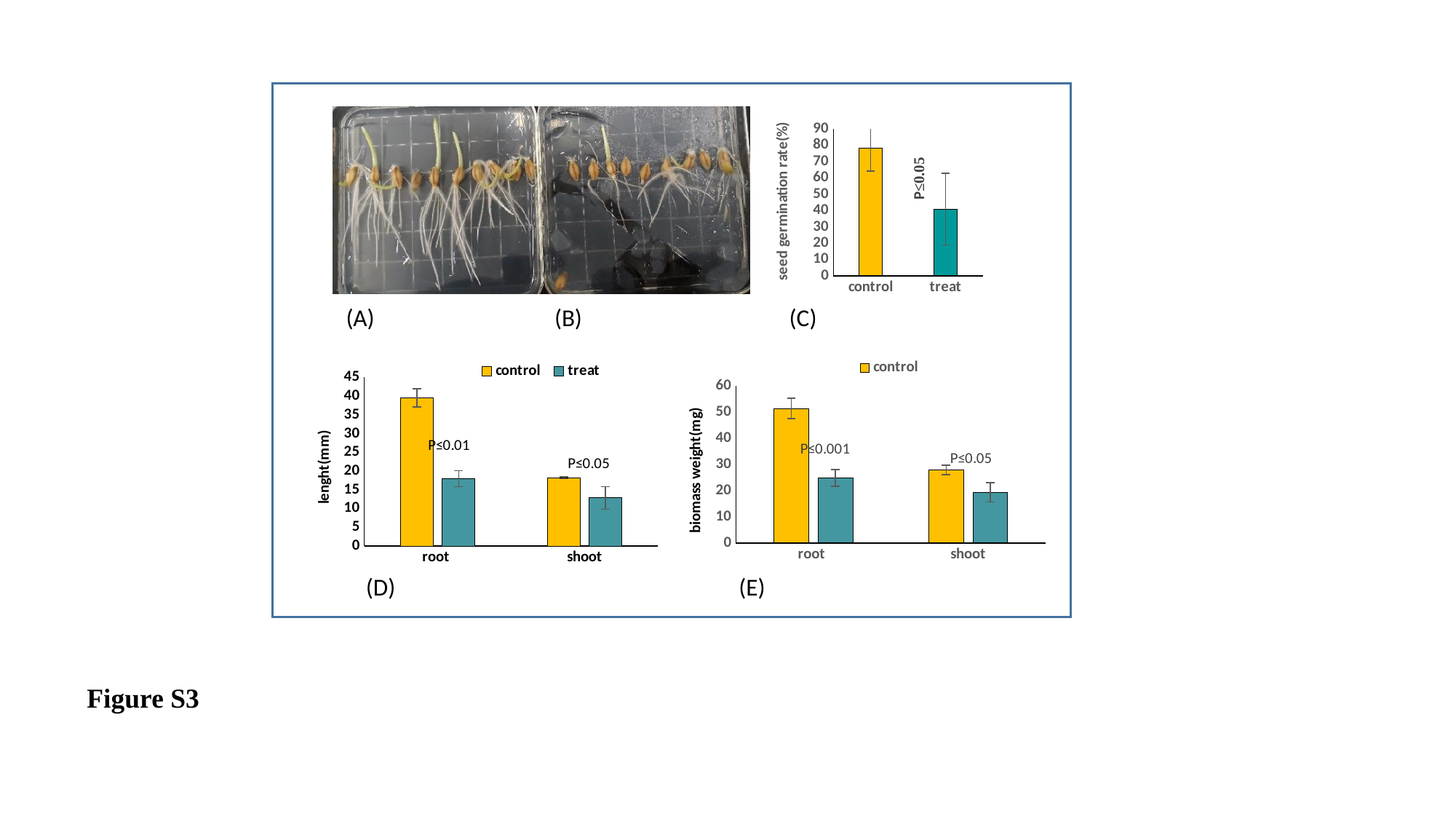

### Chart:
| Category | seed germination rate |
|---|---|
| control | 78.125 |
| treat | 40.875 |
(A) (B) (C)
### Chart
| Category | control | treat |
|---|---|---|
| root | 39.56 | 18.0 |
| shoot | 18.25 | 12.83 |
### Chart
| Category | control | treat |
|---|---|---|
| root | 51.44 | 24.84 |
| shoot | 27.86 | 19.35 | (D) (E)
Figure S3

## Slide 5
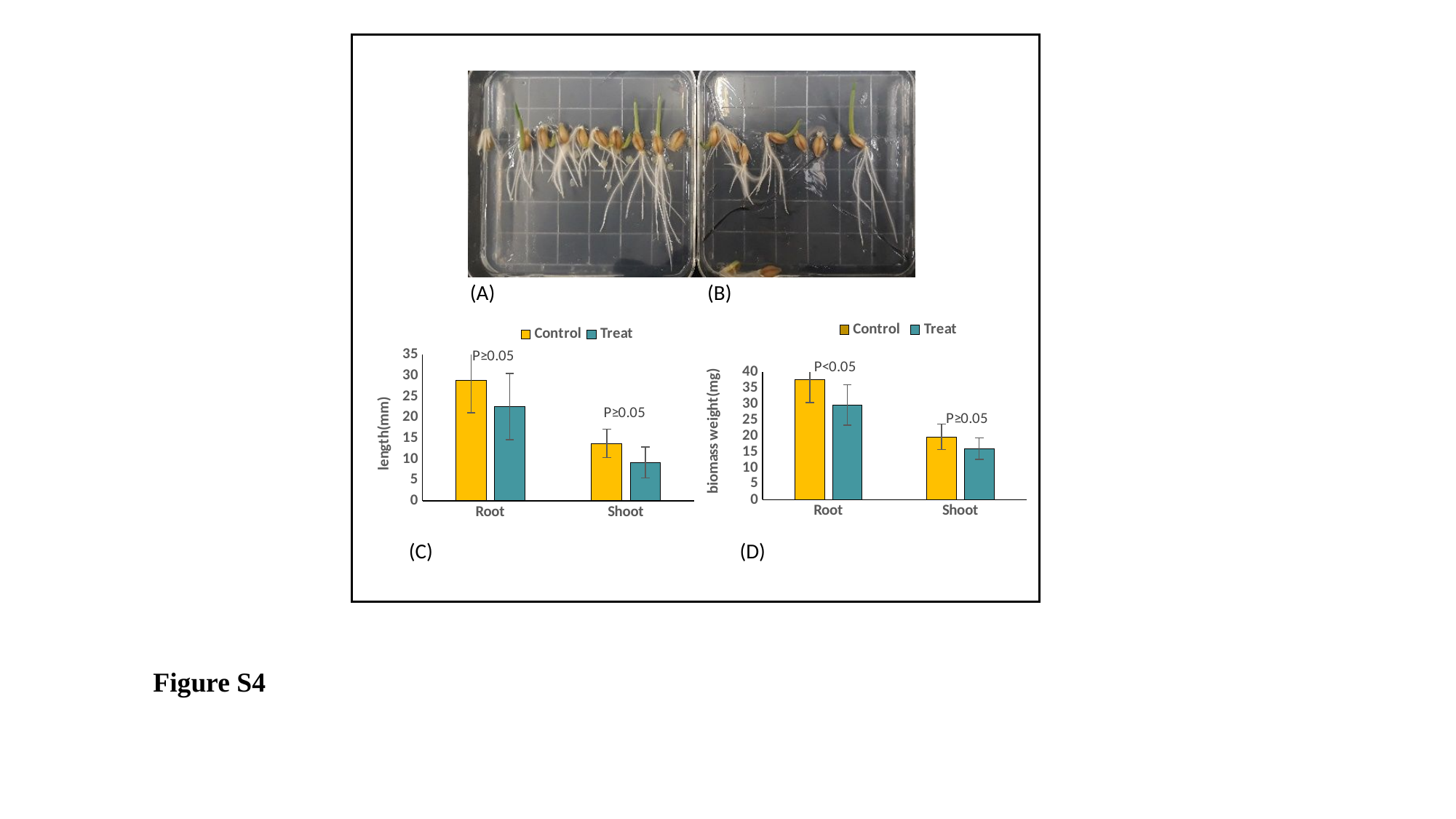

(A) (B)
### Chart
| Category | Control | Treat |
|---|---|---|
| Root | 37.61 | 29.64 |
| Shoot | 19.69 | 16.01 |
### Chart
| Category | Control | Treat |
|---|---|---|
| Root | 28.81 | 22.6 |
| Shoot | 13.72 | 9.19 |(C) (D)
Figure S4

## Slide 6
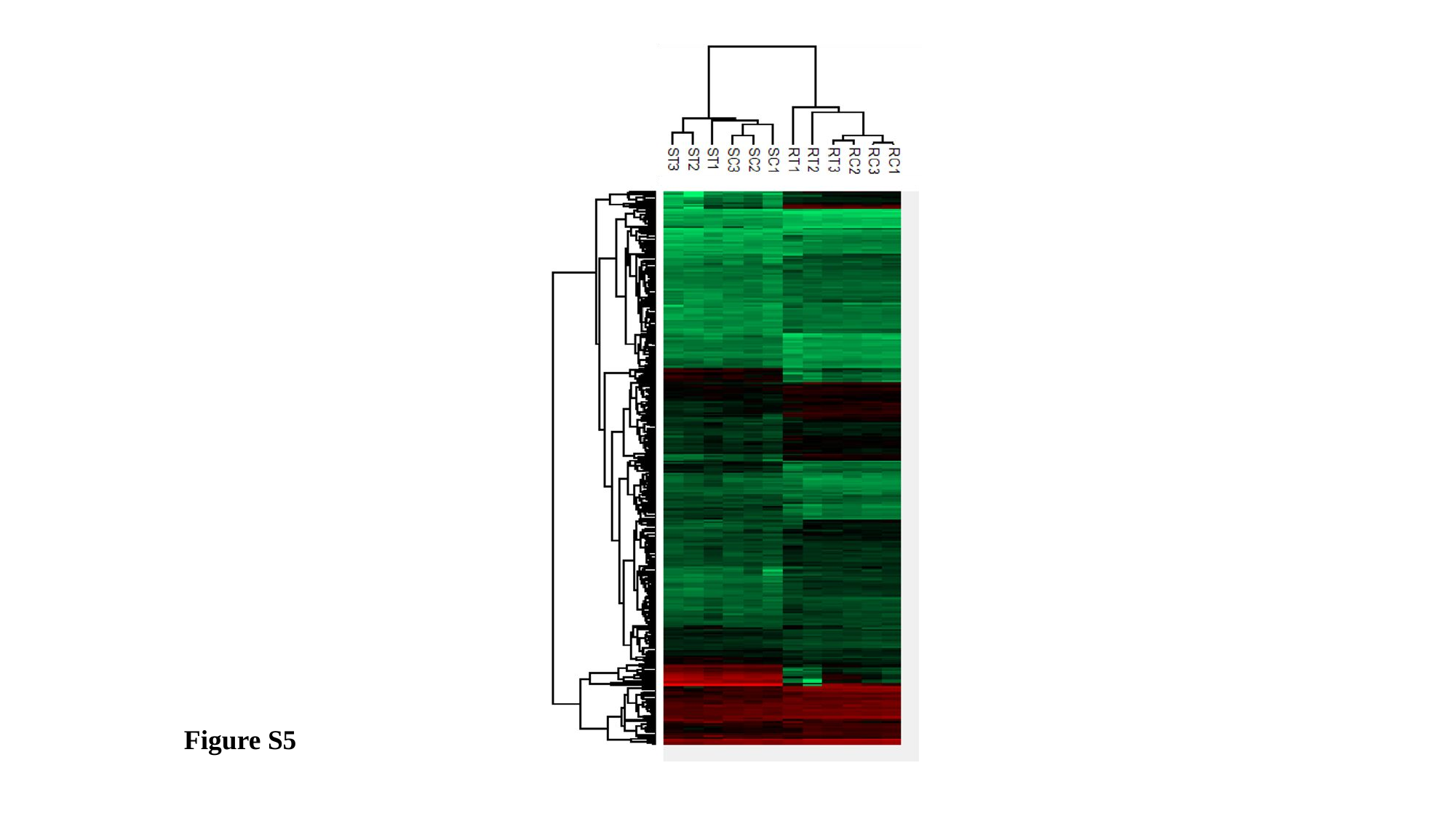

Figure S5
